# Genome Evolution and Plasticity of *Serratia marcescens*, an Important Multidrug-Resistant Nosocomial Pathogen

**DOI:** 10.1093/gbe/evu160

**Published:** 2014-07-28

**Authors:** Atsushi Iguchi, Yutaka Nagaya, Elizabeth Pradel, Tadasuke Ooka, Yoshitoshi Ogura, Keisuke Katsura, Ken Kurokawa, Kenshiro Oshima, Masahira Hattori, Julian Parkhill, Mohamed Sebaihia, Sarah J. Coulthurst, Naomasa Gotoh, Nicholas R. Thomson, Jonathan J. Ewbank, Tetsuya Hayashi

**Affiliations:** ^1^Interdisciplinary Research Organization, University of Miyazaki, Japan; ^2^Department of Microbiology and Infection Control Science, Kyoto Pharmaceutical University, Japan; ^3^Centre d’Immunologie de Marseille-Luminy, UM2 Aix-Marseille Université, Marseille, France; ^4^INSERM, U1104, Marseille, France; ^5^CNRS, UMR7280, Marseille, France; ^6^Department of Infectious Diseases, Faculty of Medicine, University of Miyazaki, Japan; ^7^Department of Genomics and Bioenvironmental Science, Frontier Science Research Center, University of Miyazaki, Japan; ^8^Earth-Life Science Institute, Tokyo Institute of Technology, Kanagawa, Japan; ^9^Graduate School of Frontier Sciences, University of Tokyo, Chiba, Japan; ^10^Pathogen Genomics, The Wellcome Trust Sanger Institute, Wellcome Trust Genome Campus, Cambridge, United Kingdom; ^11^Division of Molecular Microbiology, College of Life Sciences, University of Dundee, United Kingdom; ^12^Department of Infectious and Tropical Diseases, London School of Hygiene and Tropical Medicine, London, United Kingdom; ^13^Present address: Department of Animal and Grassland Sciences, Faculty of Agriculture, University of Miyazaki, Japan; ^14^Present address: Kashima ONC QC, Oncology DCU, Eisai Demand Chain Systems, Eisai Co., Ltd., Ibaraki, Japan; ^15^Present address: CIIL-Inserm U1019, Institut Pasteur de Lille, Lille, France

**Keywords:** *Serratia marcescens*, genome plasticity, virulence, multidrug resistance

## Abstract

*Serratia marcescens* is an important nosocomial pathogen that can cause an array of infections, most notably of the urinary tract and bloodstream. Naturally, it is found in many environmental niches, and is capable of infecting plants and animals. The emergence and spread of multidrug-resistant strains producing extended-spectrum or metallo beta-lactamases now pose a threat to public health worldwide. Here we report the complete genome sequences of two carefully selected *S. marcescens* strains, a multidrug-resistant clinical isolate (strain SM39) and an insect isolate (strain Db11). Our comparative analyses reveal the core genome of *S. marcescens* and define the potential metabolic capacity, virulence, and multidrug resistance of this species. We show a remarkable intraspecies genetic diversity, both at the sequence level and with regards genome flexibility, which may reflect the diversity of niches inhabited by members of this species. A broader analysis with other *Serratia* species identifies a set of approximately 3,000 genes that characterize the genus. Within this apparent genetic diversity, we identified many genes implicated in the high virulence potential and antibiotic resistance of SM39, including the metallo beta-lactamase and multiple other drug resistance determinants carried on plasmid pSMC1. We further show that pSMC1 is most closely related to plasmids circulating in *Pseudomonas* species. Our data will provide a valuable basis for future studies on *S. marcescens* and new insights into the genetic mechanisms that underlie the emergence of pathogens highly resistant to multiple antimicrobial agents.

## Introduction

*Serratia* species are ubiquitous in the environment, and are found in water and soil as well as associated with plants, insects, humans, and other animals. The genus *Serratia* belongs to the family *Enterobacteriaceae* and comprises at least 14 species with two subspecies (Mahlen 2011). Among these *Serratia* species, *S. marcescens* is the one most commonly associated with human infections. Originally considered as nonpathogenic, it is now recognized as an important nosocomial pathogen capable of causing urinary tract infections (Maki et al. 1973; Okuda et al. 1984; Su et al. 2003), bloodstream infections including endocarditis (Korner et al. 1994), and many other types of infections (Yu 1979). Several potential virulence factors of *S. marcescens* have been identified, including hemolysin (Goluszko and Nowacki 1989; Shimuta et al. 2009), proteases (Lyerly et al. 1981; Lyerly and Kreger 1983), lipopolysaccharide (LPS) (Makimura et al. 2007), fimbriae (Parment et al. 1992), and siderophores (Letoffe et al. 1994). The pathogenicity and genomic plasticity of members of this species are, however, yet to be fully understood.

One other important feature of *S. marcescens* as a nosocomial pathogen is its intrinsic and acquired resistance to antimicrobial agents. Many of the clinical isolates of this organism carry chromosomal and plasmid-encoded genetic determinants specifying resistance to a wide range of antibiotics (Mahlen 2011) including extended-spectrum beta-lactamase (ESBL) or metallo beta-lactamase (MBL). For example, surveys in Poland (1996–2000) in two hospitals showed 19% (67/354) of *S. marcescens* isolates produced ESBL (Naumiuk et al. 2004). Similarly in Taiwan (2001–2002), 12% (15/123) were ESBL producers and the 30-day mortality rate of patients with ESBL-producing *S. marcescens* was 33% (Cheng et al. 2006). MBL-producing *S. marcescens* are clinically more problematic because they show a high level resistance to a wider range of beta-lactams including carbapenem. A representative MBL enzyme, IMP-1, was first seen in a *S. marcescens* clinical isolate in 1991 in Japan (Osano et al. 1994). Since then various types of MBL have been identified in many *S. marcescens* strains (Wachino et al. 2011), including those causing outbreaks (Herbert et al. 2007; Nastro et al. 2013).

Here, we report and compare the complete genome sequences of two *S. marcescens* strains; a clinical isolate that showed a high level multidrug resistance (strain SM39) and a spontaneous streptomycin-resistant mutant derived from the strain Db10 originally isolated from a moribund fly (strain Db11). Our analysis reveals a possible core genome of *S. marcescens* and accessory genomes specific to each strain, providing insights into the high virulence potential of the clinical isolate. We found that the extremely high level of multidrug resistance of strain SM39 was due to the presence of the plasmid pSMC1, which encodes MBL and several other drug resistance determinants. We propose a scenario for the origin and evolution of pSMC1, based on its genomic features.

## Materials and Methods

### *S. marcescens* Strains, Culture Media, and Growth Conditions

Strain SM39 was isolated from a septicemic patient in Japan in 1999 (Nakamura et al. 2002). Strain Db11, a kind gift from Dominique Ferrandon, is a spontaneous streptomycin-resistant derivative of strain Db10 which was isolated from a moribund *Drosophila melanogaster* in Sweden (Flyg et al. 1980). It is available, together with Db10, from the *Caenorhabditis* Genetics Center (http://www.cbs.umn.edu/CGC, last accessed August 6, 2014). For routine bacterial cultivation, the strains were aerobically grown at 37 °C in Luria–Bertani (LB) broth with shaking or on LB agar plates.

### Genome Sequencing, Gene Prediction, and Annotation

Genomic DNA was prepared from overnight cultures of the two strains using the Genomic-tip 100/G and Genomic DNA buffer set (Qiagen, Inc.) according to the manufacturer’s instructions. The genome of SM39 was shotgun sequenced to approximately 12-fold coverage from 102,218 end sequences of two genomic shotgun libraries based on pUC118 and pCC1BAC with average insert sizes of 3 and 10 kb, respectively. The genome of Db11 was shotgun sequenced to approximately 10-fold coverage from 90,142 end sequences from multiple shotgun libraries: pMAQ1Sac_*Bst*XI (with insert sizes of 5.5–6, 9–10, and 10–12 kb), pUC19 (with insert sizes of 1.4–2 and 2–2.8 kb), pOTWI2 (with insert sizes of 3–3.3 and 2–2.8 kb), and M13mp18 (with insert sizes of 1–1.4, 0.4–0.8, 1–1.4, and 0.5–1 kb). This was supplemented by approximately 0.2-fold coverage from 1,713 end sequences derived from large insert fosmid libraries: pBACe3.6_*Bam*HI with an insert size 18–23 kb. All sequencing was performed using big-dye terminator chemistry on ABI3730 or ABI3700 capillary automated sequencers (Applied Biosystems). All assemblies were generated using Phrap. All repeat regions, gaps and low quality regions were bridged using large insert fosmid libraries, read-pairs, or end-sequenced polymerase chain reaction (PCR) products. The sequences were manipulated to the community standard of “Finished” (Chain et al. 2009).

Nucleotide sequence position 1 of each chromosome was assigned according to that of published *Escherichia* *coli* genomes. Gene prediction and annotation were performed using Microbial Genome Annotation Pipeline (Sugawara et al. 2009), followed by manual curation on the basis of the results of BLASTP homology search against the public nonredundant protein database (http://www.ncbi.nlm.nih.gov/, last accessed August 6, 2014). The annotated genome sequences of SM39 and Db11 have been deposited to the DDBJ/EMBL/GenBank database under the accession numbers AP013063 for the SM39 chromosome, AP013064 for the pSMC1 plasmid of SM39, AP013065 for pSMC2 of SM39, and HG326223 for the Db11 chromosome.

### Genome-Wide Comparative Analysis

Intra- and interstrain clustering analyses of protein coding sequences (CDSs) were performed based on the results of all-to-all BLASTP analysis with a threshold of ≥90% amino acid sequence identity over ≥90% aligned length of the query sequence (supplementary fig. S1, Supplementary Material online, for details). In Silico Molecular Cloning (InSilico Biology, Yokohama, Japan) was used for genome sequence comparison. The average nucleotide identity (ANI) was calculated using the JSpecies software (Richter and Rossello-Mora 2009). Multiple alignments of sequences were constructed using the ClustalW program (Thompson et al. 1994) and a neighbor-joining tree was generated using the Tamura–Nei model in MEGA5 (Tamura et al. 2007). Pathway analysis was performed using the KEGG (Kyoto Encyclopedia of Genes and Genomes) online database (Kanehisa and Goto 2000). Signal peptide-containing proteins were identified using the SignalP 4.1 server (http://www.cbs.dtu.dk/services/SignalP/, last accessed August 6, 2014) (Petersen et al. 2011). For the interspecies comparison, following four genomes were used: *S. proteamaculans* strain 568 (GenBank accession number CP000826) and *S. plymuthica* strains AS9 (CP002773) (Neupane et al. 2012) and 4Rx13 (CP006250) (Weise et al. 2014), which were all isolated from plants, and *S. odorifera* strain DSM4582 (from human sputum) (ADBY00000000).

### Semiautomated Screen for Mutants with an Attenuated Virulence Using *Caenorhabditis* *elegans*

A library of individual *S. marcescens* Db10 mini-Tn5Sm mutant clones was generated using standard methods. Using a COPAS Biosort (Union Biometrica, Boston), 30 L4 stage wild type (N2) worms were sorted into the wells of 96-well plates as previously described (Garvis et al. 2009). Then, 30 µl of an overnight culture (30 °C in 200 µl LB with 100 µg/ml streptomycin in 96-well plates) of individual mutant clones was transferred to the worm-containing wells. Assay plates containing worms and bacteria were incubated at 25 °C with agitation, whereas overnight culture plates were kept at 4 °C. A duplicate assay was performed the following day, again with 30 L4 worms per well, using the same bacterial cultures. For this, bacterial cultures were warmed up from 4 °C to room temperature for at least an hour before use. For each assay and its duplicate, the number of worms alive in each well was scored each day for 5 days by examination with a dissecting microscope. Wells were scored as − (<5 worms alive), + (5–9 alive), ++ (10–14 alive), and +++ (at least 15 alive). Mutants were considered attenuated when they scored either ++ after 3 days, or + after 4 or 5 days, and were subsequently assayed for worm killing on solid medium as previously described (Kurz et al. 2003). The mini-Tn5-Sm insertion site in attenuated mutants was determined by direct genomic DNA sequencing (MilleGen, Labège, France) with primers JEP131 and JEP132 (5′-CGGCCGCACTTGTGTATAA-3′ and 5′-CTAGGCGGCCAGATCTGATCAA-3′, respectively).

### Construction of KS3, a pSMC1-Cured Derivative of SM39

Because the pSMC1 plasmid of SM39 is highly stable, we could not obtain SM39-derivatives that have spontaneously lost pSMC1. To obtain a pSMC1-cured derivative of SM39, we first deleted the *parABC* genes (pSMC1_53-55) of pSMC1 using a method described by Masuda et al. (2000). The upstream and downstream *parABC*-flanking regions were amplified by PCR using primer pairs parAUF4/parAUR2 (5′-GGAATTCAGCTAGCTTCTAGATGACCAGAAA-3′ and 5′-CAGAGAACAACAAGATAGATTTTAGCCGCTAAA-3′) and parADF2/parADR3 (5′-GCTAAAATCTATCTTGTTGTTCTCTGTTATTCCC-3′ and 5′-CTCGAGCTCTGTGCGCATCGAGTT-3′), respectively. The PCR products were concatenated by fusion PCR with primers parAUF4 and parADR3. The concatenated DNA fragment was inserted into pLOI2223 to yield pPAR001, and then a *Not*I fragment of pMT5071, which encodes the Mob cassette, was inserted into pPAR001 to yield pPAR002. pPAR002 was mobilized conjugally from *E. coli* strain S17-1 to *S. marcescens* SM39. Transconjugants, in which pPAR002 was inserted into pSMC1 (the first recombination), were selected on BM2 plates containing 150 µg/ml of chloramphenicol (Cm) and sucrose-sensitive clones were further screened using sucrose (10%)-containing and sucrose-free LB plates. Insertion of pPAR002 into pSMC1 was confirmed by PCR, and then pPAR002 excision (with or without the *parABC* genes) was selected on sucrose LB plates. Cm-susceptible clones were screened by PCR for the loss of the *parABC* genes. SM39-derivatives cured of pSMC1 were sensitive to HgCl_2_ (16 µg/ml in LB plates). The loss of pSMC1 was finally confirmed by plasmid profiling, and one of these derivatives (designated as KS3) was used for further analysis. See supplementary figure S2, Supplementary Material online, for the entire process and the composition of BM2 medium.

### Antimicrobial Susceptibility Testing

Minimum inhibitory concentrations (MICs) of strains SM39, KS3, and Db11 against 36 antimicrobial agents were determined by the usual 2-fold agar dilution technique with Mueller-Hinton II agar (Becton Dickinson Microbiology Systems, Cockeysville, MD) with an inoculum size of 10^4^ cells as described previously (Masuda et al. 2000).

## Results and Discussion

### Comparative Genomics of Human Clinical and Insect Pathogenic *S. marcescens* Strains SM39 and Db11

**Fig. 1.—:**
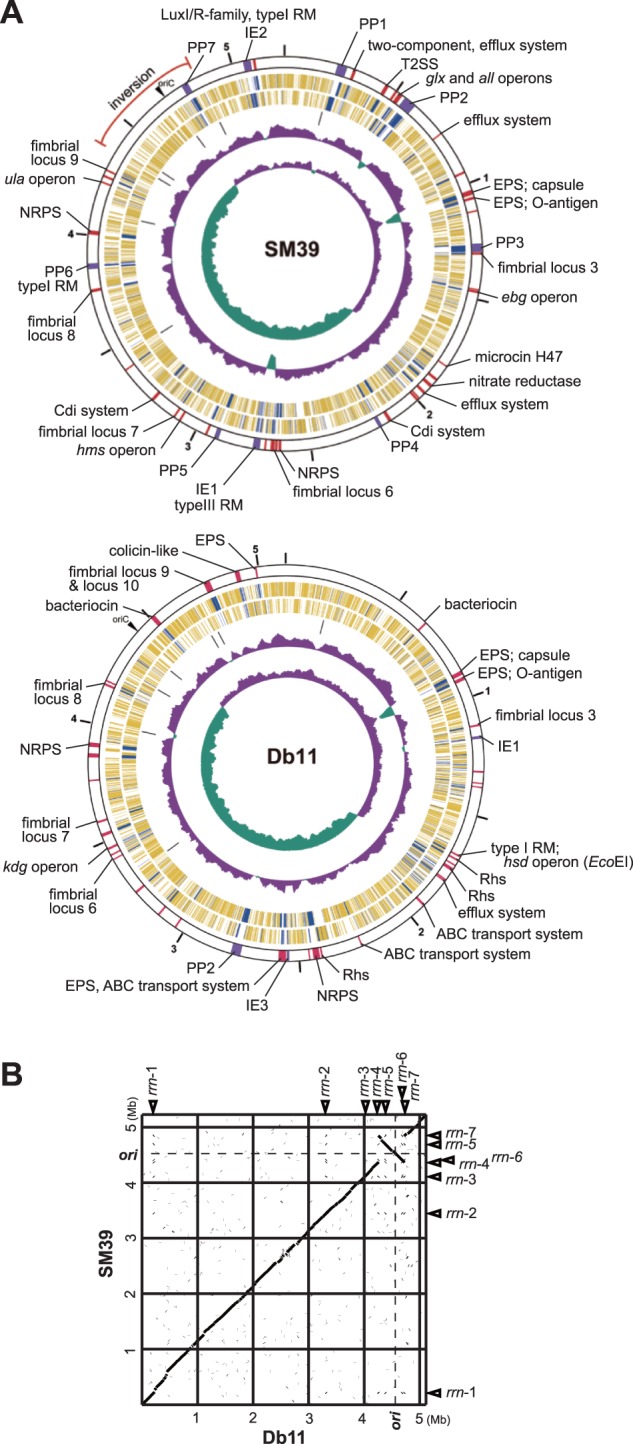
The chromosomes of *Serratia marcescens* strains SM39 and Db11. (*A*) Circular maps of the SM39 and Db11 chromosomes. From the outside in, the first circle shows the nucleotide sequence positions (in Mb), and the second circle shows the locations of strain-specific regions of ≥5 kb (purple: prophages and integrative elements; red: others) with an indication of their features and/or encoded products/functions (PP, prophages; IE, integrative elements; EPS, exopolysaccharide biosynthesis). The third and fourth circles show CDSs transcribed clockwise and anticlockwise, respectively (yellow: CDSs conserved in both strains, blue: CDSs specific to one strain), the fifth circle the rRNA operons, the sixth circle the G+C content, and the seventh circle the GC skew. (*B*) Dot plot presentation of DNA sequence homologies between the chromosomes. Locations of *ori* and seven rRNA operons (*rrn1–rrn*7) are indicated.

The chromosomes of SM39 and Db11 are similar in size, 5,225,577 and 5,113,802 bp, respectively, and gene number (table 1 and fig. 1*A*). SM39 carries two plasmids, pSMC1 (41,517 bp) and pSMC2 (58,929 bp). No extrachromosomal elements were found in Db11. Overall, the chromosomes of the two strains are highly conserved and essentially collinear, except for a large inversion of the *oriC* region flanked by rRNA operons (fig. 1*B*). Surprisingly for members of the same species, the ANI between the conserved genomic regions of the two strains (4,391 kb) is only 95.1%. This can be compared with a figure of 97.0% for the ANI between two distantly related members of *E. coli*, strains K-12 and E2348/69. Despite this, the 16S rRNA genes of the two strains show 99.4% sequence identity, consistent with DB11 and SM39 being members of the same species (Kim et al. 2014; see also supplementary fig. S3, Supplementary Material online). Further with a criterion of ≤90% nucleotide sequence identity and ≥5 kb length, we identified 44 genomic regions specific to SM39, totaling 628 kb (12% of the genome), and 39 specific regions (447 kb or 8.7% of the genome) for Db11 (fig. 1*A*; see also supplementary tables S1 and S2, Supplementary Material online). The relatively low ANI value for the two *S. marcescens* strains and the considerable amount of isolate-specific sequences may reflect the diversity of niches that this species can occupy.

**Table 1:** Genomic Features of *Serratia marcescens* Strains SM39 and Db11

Strains	SM39		Db11
Chromosome				
Size (bp)	5,225,577		5,113,802
GC content (%)	59.8		59.5
CDSs^a^	4,866 (19)		4,722 (15)
rRNA operons	7		7
tRNAs	87		87
Prophages	7		2
Integrative elements	2		4
IS elements	17		5

Plasmid s	pSMC1	pSMC2	None

Size (bp)	41,517	58,929	—
GC content (%)	61.5	51.9	—
CDSs^a^	55 (4)	72 (0)	—
IS elements	1	0	—

^a^The number of pseudogenes is indicated in parentheses.

**Fig. 2.—:**
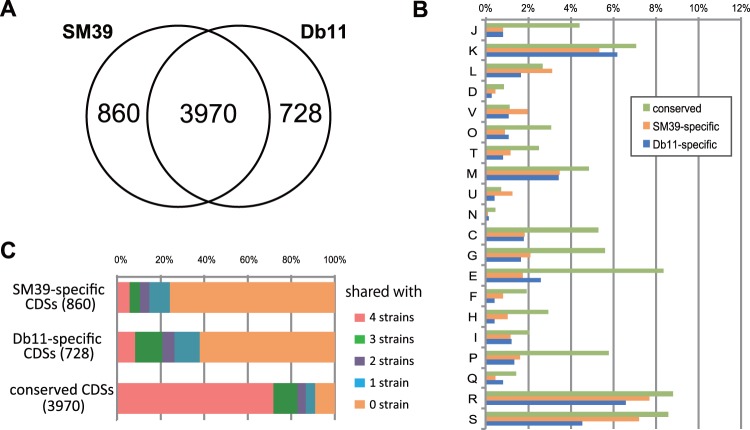
Comparison of the gene contents of SM39 and Db11. (*A*) Venn diagram showing the numbers of conserved and strain-specific CDSs. (*B*) COG category-based functional analysis of each group of CDSs, the conserved and strain-specific CDSs. J: translation, ribosomal structure, and biogenesis; K: transcription; L: replication, recombination, and repair; D: cell cycle control, cell division chromosome partitioning; V: defense mechanisms; O: posttranslational modification, protein turnover, and chaperones; T: signal transduction mechanisms; M: cell wall/membrane/envelope biogenesis; U: intracellular trafficking, secretion, and vesicular transport; N: Cell motility; C: energy production and conversion; G: carbohydrate transport and metabolism; E: amino acid transport and metabolism; F: nucleotide transport and metabolism; H: coenzyme transport and metabolism; I: lipid transport and metabolism; P: inorganic ion transport and metabolism; Q: secondary metabolites biosynthesis, transport, and catabolism; R: general function prediction only; S: function unknown. (*C*) Conservation of each group of CDSs in four strains of other *Serratia* species (*S. proteamaculans* 568, *S. odorifera* DSM4582, *S. plymuthica* 4Rx13, and *S. plymuthica* AS9).

To extend the analysis of unique and shared genes between the two strains, we performed a clustering analysis of the SM39 and Db11 CDSs using an all-against-all BLASTP approach (outlined in supplementary fig. S1, Supplementary Material online). This showed that 3,970 genes (or gene families) were conserved in both strains with additional 860 SM39-specific and 728 Db11-specific genes (fig. 2*A*). Functional classification of these genes based on the Cluster of Orthologous Groups (COG) categories indicated that genes belonging to “category L (replication, recombination, and repair)” and “category V (defense mechanisms)” are more highly represented in SM39 than in Db11 (fig. 2*B*). These differences are largely attributable to the presence of more genes associated with lateral gene transfer in SM39 such as insertion sequence (IS) transposases and integrases (category L), and genes for restriction-modification systems and multidrug transport systems (category V). For example, SM39 carries a higher variety and number of IS elements: 18 compared with the 5 carried by Db11 (table 1). These IS elements were classified into 11 distinct types, nine of which represent novel IS elements. Eight types were found in SM39 and four in Db11, with only IS*Se5* being shared by both isolates (supplementary table S3, Supplementary Material online). More prophages were also found in SM39 (seven) than in Db11 (only two), with none of the prophages and other integrative elements being shared by the two strains (table 1 and supplementary table S4, Supplementary Material online).

We further compared the *S. marcescens* gene sets with those of four sequenced strains from other *Serratia* species: *S. proteamaculans* strain 568 and *S. plymuthica* strains AS9 (Neupane et al. 2012) and 4Rx13 (Weise et al. 2014), which were all isolated from plants, and *S. odorifera* strain DSM4582 (from human sputum). This showed that only 23% and 38% of the genes that we denoted as SM39- or Db11-specific had homologs in these isolates representing other *Serratia* species (fig. 2*C*). In contrast, 71% (2,852 genes) of the 3,970 core genes shared by the two *S. marcescens* strains were conserved in all the four isolates representing other *Serratia* species. This suggests that, despite the genetic diversity of this genus (see supplementary fig. S3, Supplementary Material online, for the phylogenetic relationship of these species in genus *Serratia*), there is a relatively large shared or core genome of 2,852 CDSs. This analysis also identified 358 genes that are conserved in the two *S. marcescens* strains but absent in other *Serratia* species. This group includes a number of genes related to pathogenicity in other bacteria (data not shown).

### Common and Strain-Specific Metabolic Capabilities in *S. marcescens* SM39 and Db11

Central metabolic pathways are well conserved between the two strains and characteristic of the species as a whole: Positive fermentation for sucrose and d-sorbitol, and negative for d- and l-arabinose, l-rhamnose, d-xylose and cellubiose. Not uncommonly, both isolates lack the genes for prodigiosin biosynthesis, the characteristic red pigment often associated with *S. marcescens* (Harris et al. 2004). The differences between the two isolates include, for carbohydrate metabolism, alternative pathways for l-ascorbate utilization; the *ula*-type encoded pathway is carried by SM39 (SM39_4088-4095) and the *sgb-*type by Db11 (SMDB11_3334-3337). Major differences in nitrogen metabolism are also observed between the two isolates: Unlike Db11, SM39 carries the *nrtABC* operon (SM39_1878-1880) encoding an ATP-binding cassette (ABC) transporter for nitrate/nitrite, the *nasAC* operon (SM39_1885 and SM39_1886) encoding a nitrate reductase, and the *nasB* gene (SM39_1884) encoding a nitrite reductase (all in the SM39_E18 region; supplementary table S1, Supplementary Material online). Thus SM39 appears to have a potential capacity to utilize nitrate, which is present in urine, as a nitrogen source. Related to this, both SM39 and Db11 carry an operon necessary for the metabolism of allantoin but lack the *allB* gene encoding allantoinase (Cusa et al. 1999). Allantoin is the principal nitrogen source in urine in most mammals. Humans and nonhuman primates, however, carry a genetic lesion, thus rather than allantoin, they accumulate urate that can be catabolized despite the loss of *allB*. Together, these genes could be associated with the potential capacity of *S. marcescens* strains to grow in urine and cause urinary tract infections in humans (Mahlen 2011).

### Diversity and Differential Evolution of the Potential Virulence of *S. marcescens* SM39 and Db11

*Serratia* *marcescens* is well known for its ability to secrete numerous exoenzymes and other proteins. We identified many known or predicted secreted or surface-exposed proteins potentially related to virulence, most of which (30/40) are conserved in both strains (supplementary table S5, Supplementary Material online).

#### Surface Structures and Polysaccharides

Here again, the genomic analysis revealed conservation and specialization of the outer surface of the two sequenced strains. *Serratia* *marcescens* is characteristically motile, and both strains carry a complete *E. coli-*like gene set for the biosynthesis of flagella and for chemotaxis (supplementary table S6, Supplementary Material online). These gene sets are highly conserved between SM39 and Db11, the exception being the *fliC* flagellin-encoding gene, which shows a remarkable sequence diversity (only 68.3% identity in the amino acid sequence). Variation in flagellin structure is linked with antigenic variation and differences in the helicity of the flagellum that in turn are associated with niche adaptation.

**Fig. 3.—:**
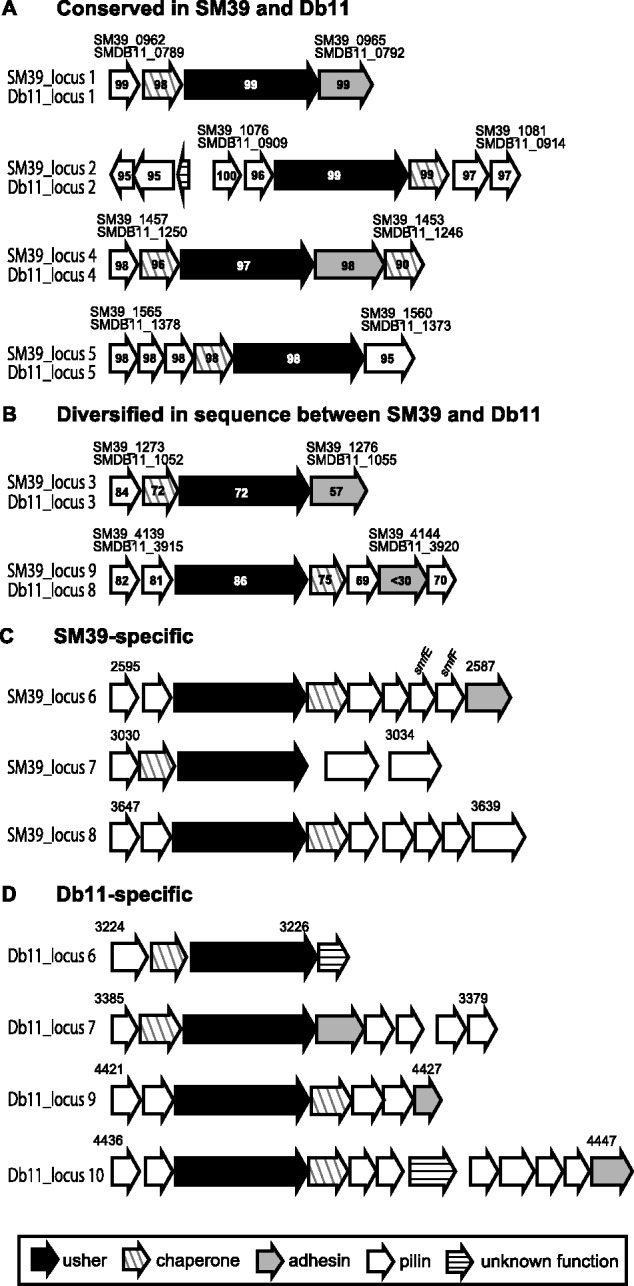
Fimbriae operons identified in SM39 and Db11. The gene organization of the operons for biosynthesis of chaperone-usher fimbriae identified in SM39 and Db11 is shown. (*A*) Operons “conserved” between the two strains showing a high sequence identity (>95% amino acid sequence identity for all gene products) and (*B*) genes in the “diversified” operons show low sequence identity (up to 86% amino acid sequence identity). Note that both strains contain a set of type IV fimbriae-related genes, orthologs of which have been identified of *Escherichia coli,* and that a homologue (SM39_0944) of *yagZ*/*ecpA*/*matB*, the gene for *E. coli* common pili (also known as Mat fimbriae) was found in SM39 but not in Db11.

Both Db11 and SM39 possess multiple operons for the biosynthesis of chaperone–usher fimbriae associated with biofilm formation and attachment to biotic and abiotic surfaces (Labbate et al. 2007; Shanks et al. 2007). Among these, six are conserved in the two strains (two are significantly divergent in sequence), but three and four are specific to SM39 and Db11, respectively (fig. 3). In addition, although both strains contain a complete set of type IV fimbriae-related genes (Sauvonnet et al. 2000; Kulkarni et al. 2009; Xicohtencatl-Cortes et al. 2009) (supplementary table S7, Supplementary Material online), only SM39 contains a homolog (SM39_0944) of *yagZ*/*ecpA*/*matB*, the gene for *E. coli* common pili (also known as Mat fimbriae) (Pouttu et al. 2001; Rendon et al. 2007).

Although the SM39 and Db11 LPS core polysaccharide biosynthesis gene clusters (the *waa* genes) are broadly similar, there is significant sequence divergence (25–71% amino acid sequence identity for the corresponding predicted proteins) in four orthologous genes (SM39_4551-4554 and SMDB11_4052-4055) in this cluster. These four genes encode two glycosyltransferases, a polymer ligase (WaaL), and an UDP-galactose-4-epimerase. Those of Db11 showed high similarity to those of *S. marcescens* strain N28b serovar O4 (Coderch et al. 2004). These data suggest that at least two LPS core types exist in *S. marcescens.*

**Fig. 4.—:**
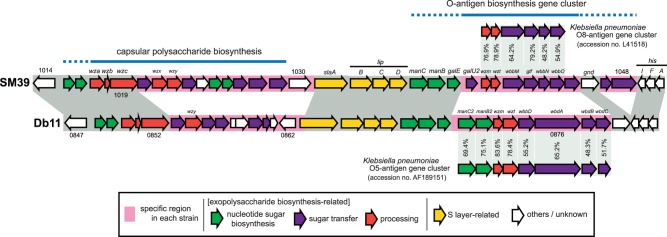
Comparison of the SM39 and Db11 genomic loci bearing exopolysaccharide biosynthesis gene clusters. The gene organization of the gene clusters for O antigen biosynthesis and for group 1 CPS biosynthesis is compared between SM39 (untypeable) and Db11 (O28:K7). The O antigen biosynthesis genes of SM39 and Db11 show high level of similarities to those of *Klebsiella pneumoniae* O8 and those of *K. pneumoniae* O5, respectively. The Db11 operon also has a high level of similarity to that of *Escherichia coli* O8 (Iguchi A, Iyoda S, Kikuchi T, Ogura Y, Katsura K, Ohnishi M, Hayashi T and Thomson NR, unpublished data), consistent with the cross-reactivity between *Serratia marcescens* O28*, K. pneumoniae* O5, and *E. coli* O8 antigens previously reported by Aucken and Pitt (1991).

The gene cluster for O antigen biosynthesis was identified between the S-layer biosynthesis gene cluster and the *his* operon in both strains (fig. 4). As expected, the gene contents of the loci significantly differ between the two strains; Db11 was serotyped conventionally as O28:K7, whereas SM39 was untypeable. There is also good evidence from the genome sequences that both strains carry a complete gene cluster for group 1 capsule polysaccharide (CPS) biosynthesis, equivalent to the colanic acid biosynthesis genes in *E. coli.* Differences in gene content in this region suggest, however, that the two strains produce different types of group 1 CPS (fig. 4). This will need to be addressed experimentally in future studies.

#### Secretion Systems and Secreted Proteins

One unexpected difference between Db11 and SM39 is the presence of a type II secretion system (T2SS) in SM39 but not in Db11 (supplementary fig. S4, Supplementary Material online). The T2SS is a multiprotein secretion complex, present in a wide variety of organisms and frequently implicated in virulence (Korotkov et al. 2012). Although the substrates of the SM39 T2SS are unknown, it could well contribute to the virulence of SM39.

**Fig. 5.—:**
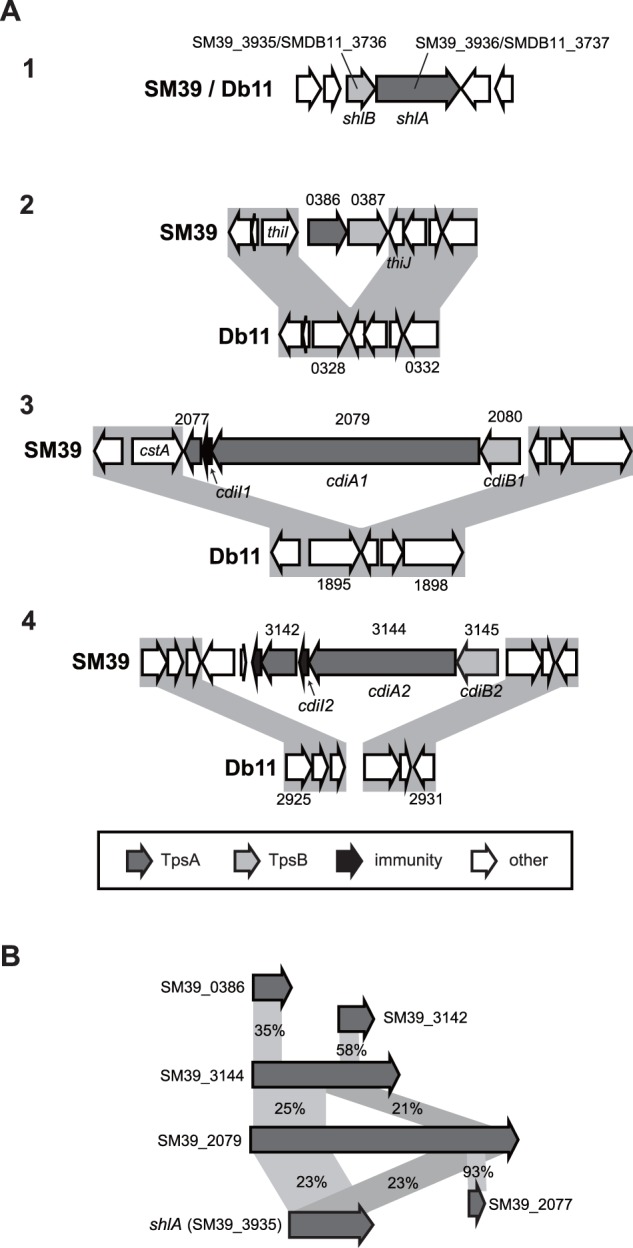
Gene clusters for hemolysin/hemagglutin-like two-partner Type V secretion systems identified in SM39 and Db11. (*A*) The gene organization of the gene clusters for hemolysin/hemagglutin-like two-partner systems in SM39 and Db11 is shown. TpsA components are the passenger proteins (including the ShlA hemolysin and CdiA proteins) and TpsB components are the cognate translocator proteins (including ShlB and CdiB proteins). Although the *shlBA* operon (image 1) is conserved in the two strains, three additional gene clusters were found only in SM39. Two of these, SM39_2080-2077 (image 3) and SM39_3145-3141 (image 4), based on homology, encode Cdi systems. In addition to CdiA and CdiB proteins, a third component conferring resistance to the C-terminal toxin domain of CdiA is encoded downstream of CdiA, the CdiI immunity protein (SM39_2078 and SM39_3143, respectively). A putative “orphan” CdiA C-terminus (including a distinct potential toxin domain) and cognate CdiI pair is encoded by SM39_3142-3141. The function of the third SM39-specific cluster, SM39_0386-0387 (image 2), is unknown. (*B*) Similarities between the TpsA hemolysin/hemagglutin-related proteins (amino acid sequence identity) are shown, with those newly identified in SM39 showing partial similarity to ShlA.

Multiple type V secretion systems (T5SSs) are present in SM39 and Db11. T5SSs include both autotransporters and two-partner systems (Grijpstra et al. 2013; Jacob-Dubuisson et al. 2013). An archetypal example of a two-partner T5SS is ShlBA; the hemolysin ShlA is one of the known major virulence factors in *S. marcescens* (Goluszko and Nowacki 1989). The *shlBA* operon is conserved in both strains. In contrast, three additional gene clusters, encoding two-partner systems with partial similarity to ShlA and other haemagglutinin-like proteins, were identified only in SM39 (fig. 5). Two of these, SM39_2080-2077 and SM39_3145-3141, appear to encode contact-dependent inhibition (Cdi) systems. In such systems, CdiB is a translocator protein that assembles the large, hemagglutinin domain-containing CdiA passenger protein on the cell surface (Aoki et al. 2010). Variable toxin domains are found at the C-terminus of CdiA (CdiA_Ct_), which mediate contact-dependent growth inhibition of competitor bacteria. Additionally, immunity proteins (CdiI) cognate to the CdiA_Ct_ are encoded downstream of CdiA. Both loci contain a typical *cdiBAI* arrangement. The SM39_3144 locus also includes an “orphan” CdiA_Ct_–CdiI paizr (SM39_3142-3141). Orphan CdiA_Ct_–CdiI pairs contain truncated CdiA C-termini thought to represent remnants or reservoirs of alternative toxin domains, which can be exchanged to provide new antibacterial capability (Poole et al. 2011).

Both SM39 and Db11 contain Lip (lipase) and Has (hemophore) type I secretion systems (Kanonenberg et al. 2013), and both possess a single type VI secretion system (T6SS) conserved between the two strains (supplementary fig. S5, Supplementary Material online). T6SS can be used by bacteria either to target eukaryotic cells as a direct virulence mechanism or to kill rival bacterial cells as a competitive fitness mechanism (Coulthurst 2013). The *S. marcescens* T6SS has been shown to have potent bacterial killing activity (Murdoch et al. 2011). Furthermore, six effectors (antibacterial toxins) secreted by this system have recently been identified in Db11, together with cognate immunity proteins that protect against self-toxicity (English et al. 2012; Fritsch et al. 2013). Of these, the Ssp1 and Ssp2 effectors, Tae4-family peptidoglycan amidases which attack the target cell wall, and associated Rap (Tai4) immunity proteins are encoded within the T6SS gene cluster (supplementary fig. S5, Supplementary Material online). Overall, the T6SS gene clusters of Db11 and SM39 are very similar with some significant differences: Although the Ssp1–Rap1a effector–immunity pair is conserved, the “orphan” Rap1b immunity protein is missing from SM39 and Ssp2 has been replaced by a new effector–immunity pair, belonging to the same Tae4–Tai4 family but clearly distinct. This strongly suggests that the T6SS antibacterial activity of *S. marcescens* is highly specific and strain-dependent, as has been observed experimentally (Murdoch et al. 2011).

#### Iron Uptake

Iron uptake is essential for bacterial growth within host organisms because iron is sequestered by the host (Andrews et al. 2003). SM39 and Db11 possess multiple common sets of iron acquisition systems with only a few differences. Although some of the common systems are also found in other sequenced *Serratia* species (supplementary table S8, Supplementary Material online), others, including a second gene cluster for siderophore synthesis, *fecIRA* and *fepBGDC* for transport of ferric compounds, and the *has* operon, are restricted to *S. marcescens*. These species-specific iron uptake systems might be associated with the higher virulence of *S. marcescens* compared with other species of the genus.

#### Quorum-Sensing System

Quorum-sensing systems sense bacterial cell density and play important roles in regulating the behavior of bacterial population by controlling various biological processes, including pathogenicity. None of the three *N*-acyl homoserine lactone (AHL) quorum-sensing systems so far described in strains of *S. marcescens* (SwrI/SwrR [Eberl, Christiansen et al. 1996; Eberl, Winson et al. 1996], SpnI/SpnR [Horng et al. 2002], and SmaI/SmaR [Coulthurst et al. 2006]) was found in SM39 or Db11. Instead, we identified a candidate SM39-specific AHL system comprising LuxI/LuxR family proteins (SM39_4838 and SM39_4837), which show a high similarity (about 80% amino acid sequence identity) to ExpR/ExpI of *Erwinia tasmaniensis* (Muller et al. 2011). In contrast, no AHL quorum-sensing system was found in Db11. These data indicate a remarkable variation in the AHL quorum-sensing system among *S. marcescens* strains.

### Variation of Nonribosomal Peptide Synthetase-Dependent Secondary Metabolites and Weapons for Interbacterial Competition

Diffusible inhibitory molecules include antibiotics and bacteriocins (protein or peptide toxins targeting related organisms). SM39 and Db11 have one and two bacteriocins, respectively, not shared by the other, as well as two in common (including bacteriocin 28b).

An operon of six genes specific to Db11 (SMDB11_2293-2288, named *alb1–alb6*) encodes a hybrid nonribosomal peptide synthetase (NRPS)-polyketide synthase (PKS) enzyme, plus tailoring enzymes and a resistance-conferring efflux pump. This cluster was recently identified as required for the ability of Db10 to produce a diffusible antimicrobial compound and shown to direct the synthesis of a broad-spectrum antibiotic called althiomycin (Gerc et al. 2012). Two predicted NRPS enzymes are found only in SM39 and may also be involved in biosynthesis of antimicrobial molecules. These variations, along with those in T6SS and Cdi systems, suggest that interbacterial competition in microbial communities is one of the important drivers of genomic diversity in *S. marcescens*.

NRPS-dependent molecules also have other roles. In Db11, SMDB11_3680, or SwrA, produces the biosurfactant Serrawettin W2 (Pradel et al. 2007). In SM39, SM39_3884 shares only 56% identity with SwrA but, given that it is encoded in the same genomic locus and has a very similar domain organization, it probably synthesizes a related biosurfactant molecule. Three other NRPSs are shared by both strains, including two involved in synthesis of the two siderophores mentioned earlier.

### In Vivo Screening Shows That Many Core *S. marcescens* or *Serratia* sp. Genes Are Involved in Virulence in an Invertebrate Model Host

Db10 was isolated from *D.* *melanogaster* (Flyg et al. 1980), but it is pathogenic in several other infection models, including *C. elegans* and mice. Previous work has shown that there is a substantial overlap between the genes required for full virulence of *S.* *marcescens* during infection of flies and *C.* *elegans* (Kurz et al. 2003). Thus, as the nematode is well-suited to automated screens (Kurz and Ewbank 2007; Garvis et al. 2009), we used it to look for new *S.* *marcescens* virulence factors and to determine whether genes associated with virulence were within the shared or accessory gene sets. We constructed a mini-Tn*5*-Sm transposon mutant library in Db10 and assayed clones in a high-throughput screen to identify mutants with a reduced ability to kill *C. elegans*. From the 12,480 individual Db10-derived clones that were tested, 12 mutants (0.1% of all the mutants screened) were selected as showing the most robust reduction in virulence.

The mini-Tn*5*-Sm insertion site in each mutant was identified by sequencing and mapping to the genomic sequence of *S. marcescens* Db11 (for a complete list, see supplementary table S9, Supplementary Material online). Of the 12 mutants, four loci had also been identified in a previous solid medium-based screen (Kurz et al. 2003). These include those involved in O antigen biosynthesis and siderophore production. The availability of the complete sequence combined with recent findings (Murdoch et al. 2011; English et al. 2012; Fritsch et al. 2013) revealed a hitherto unsuspected contribution of a T6SS to the pathogenic capability of *S.* *marcescens*, either directly or by providing a competitive fitness advantage against other bacteria in vivo. The previously isolated mutants 22D9 and 7D1 (Kurz et al. 2003) were found to correspond to SMDB11_2265 and SMDB11_2266, respectively, which are both T6SS immunity proteins, 8C7 to SMDB11_1112, a T6SS effector protein of unknown function, whereas the previously uncloned 23C11 corresponded to SMDB11_3455, a T6SS-related, minor Hcp homolog (English et al. 2012; Fritsch et al. 2013). As previously reported, this screen also led to the isolation of a mutant in the *swrA* gene responsible for the biosynthesis of the surfactant Serrawettin W2 (Pradel et al. 2007). Additional mutants of interest include one containing an insert in a *wza* homolog, potentially involved in capsule biosynthesis, and three deficient in respiration (JESM266, JESM268, JESM271; supplementary table S9, Supplementary Material online). None of these latter mutants showed a defect in growth under standard conditions. This raises the possibility that the nematode intestine represents a restrictive respiratory environment.

The combined results of this and the previously published screens (Kurz et al. 2003) identified 30 loci important for the in vivo virulence of Db10 and/or Db11. Among the 30 genes, 22 are conserved in SM39. Of these, 11 are conserved in all of the four other *Serratia* strains analyzed (supplementary table S9, Supplementary Material online). The proportions of virulence genes conserved between Db11 and SM39 (73%) and between this common set and the other *Serratia* strains (50%) are markedly lower than the equivalent proportions of the entire gene sets (84.5% and 71%, respectively). This presumably reflects the different tropisms of the different species and the host-specific nature of many virulence factors.

### Origins of Antimicrobial Resistance in SM39 and Db11

#### Intrinsic Resistance

A large number of genes related to antimicrobial resistance are encoded on the chromosomes of both Db11 and the clinical *S. marcescens* isolate SM39. As listed in table 2, we identified many efflux pumps belonging to five families: The ABC superfamily, the major facilitator superfamily (MFS), the multidrug and toxic-compound extrusion (MATE) family, the small multidrug resistance (SMR) family, and the resistance nodulation division (RND) family. Among these, only *smdAB* (Matsuo et al. 2008), *sdeAB* (Kumar and Worobec 2005), *sdeXY* (Chen et al. 2003), *smfY* (Shahcheraghi et al. 2007), and *ssmE* (Minato et al. 2008) had been characterized in previous studies. Most of the efflux pumps are shared by the two strains although a few are strain-specific. In addition, a class C beta-lactamase AmpC, two aminoglycoside acetyltransferases each having distinct substrate specificities, and a fosfomycin-inactivating enzyme (FosA) are encoded on the chromosomes of the two strains (table 3). Thus, the overall repertoire of the chromosomally encoded resistance genes is largely similar in both strains despite their different origins, and is highly conserved accounting for the high and broad intrinsic resistance of *S. marcescens* (table 3). One notable difference between the two strains is a mutation in the *gyrA* gene of the clinically isolated SM39, generating a S83R substitution and conferring resistance to quinolone (Weigel et al. 1998).

**Table 2:** Drug Efflux Pumps Found in the Genomes of SM39 and Db11

SM39 (SM39_)	Db11 (SMDB11_)	Gene	IMP (SM39_/SMDB11_)	MFP (SM39_/SMDB11_)	OMP
ABC type					
0414-0415	0354-0355	*smdAB*	*smdA, smdB*	—	—
1134-1135	0964-0965	*macAB*	*macB*	*macA*	—
1329-1331	1118-1120	*etsABC*	*etsB*	*etsA*	*etsC*
4783-4785	4552-4554	—	4784/4553	4783/4552	—
			4785/4554	—	—
RND type					
0448-0449	0369-0370	*sdeXY*	*sdeY*	*sdeX*	—
1281-1282	1059-1060	—	1281/1059	1282/1060	—
1400-1401	1196-1197	*sdeAB*	*sdeB*	*sdeA*	—
Not found	1254A-1255A	—	SMDB11_1254A	SMDB11_1255A	SMDB11_1256
1913-1915	Not found	—	SM39_1914	SM39_1913	SM39_1915^a^
1920-1922	1741-1743	—	1921/1742	1922/1743	SMDB11_1741^a^
Not found	1698-1699	—	SMDB11_1699	SMDB11_1698	—
1958	Not found	—	SM39_1958	—	—
3100	2891	*acrD*	*acrD*	—	—
3162-3164	2945-2947	*sdeCDE*	*sdeD, sdeE*	*sdeC*	—
MFS type					
467	390	*fsr*	*fsr*	—	—
1351	1140	*mdtG*	*mdtG*	—	—
1810	1629	—	1810/1629	—	—
Not found	1759	*tetA*	*tetA*	—	—
2164	1961	*smfY*	*smfY*	—	—
2273	2069	*mdtH*	*mdtH*	—	—
2819	2580	*bcr*	*bcr*	—	—
3165	2948	*mdtD*	*mdtD*	—	—
3343-3344	3133-3134	*emrAB*	*emrB*	*emrA*	SMDB11_3132^a^
4622	4024	*emrD*	*emrD*	—	—
4391	4201	*mdfA*	*mdfA*	—	—
4497	4107	—	4497/4107	—	—
MATE type					
1653	1463	*mdtK*	*mdtK*	—	—
2598	2377	—	2598/2377	—	—
3912	3713	*dinF*	*dinF*	—	—
SMR type					
2035	1855	*ssmE*	2035/1855	—	—
2237-2238	2032-2033	*mdtJI*	2237/2032	—	—
			2238/2033	—	—
4693	4462	*sugE*	4693/4462	—	—
pSMC1_35	Not found	*qacE1*	*qacE1*	—	—
pSMC1_46	Not found	*qacE2*	*qacE2*	—	—

^a^Similar to the NodT family protein but contains no membrane-spanning domain.

**Table 3:** Drug Resistance Profiles and Genetic Determinants Responsible for Each Resistance in Strains SM39, KS3, and Db11

Antimicrobial Agents (Subclasses)	MIC (mg/ml)					Putative Resistance Determinant								
	SM39	KS3^a^		Db11			On Choromosome						On pSMC1	
						Gene			SM39/KS3 (SM39_)		Db11 (SMDB11_)		Gene	
β-Lactams														
Piperacillin (PEN)	8	2		2		*ampC*			1721		1530		*bla*_CMY_*, bla*_IMP_	
Faropenem (PEM)	>256	4		4		*ampC*			1721		1530		*bla*_CMY_*, bla*_IMP_	
Cephalothin (1st CEP)	>256	>256		>256		*ampC*			1721		1530		*bla*_CMY_*, bla*_IMP_	
Cefuroxime (2nd CEP)	>256	>256		>256		*ampC*			1721		1530		*bla*_CMY_*, bla*_IMP_	
Cefmetazol (2nd CEP)	>256	32		16		*ampC*			1721		1530		*bla*_CMY_*, bla*_IMP_	
Cefotaxime (3rd CEP)	>256	0.5		1		—			—		—		*bla*_CMY_*, bla*_IMP_	
Ceftazidime (3rd CEP)	256	0.25		1		—			—		—		*bla*_CMY_*, bla*_IMP_	
Ceftriaxone (3rd CEP)	>256	0.5		0.25		—			—		—		*bla*_CMY_*, bla*_IMP_	
Latamoxef (3rd CEP)	>256	0.25		0.125		—			—		—		*bla*_CMY_*, bla*_IMP_	
Cefbuperazone (3rd CEP)	>256	0.5		0.5		—			—		—		*bla*_CMY_*, bla*_IMP_	
Cefotetan (3rd CEP)	>256	0.5		0.5		—			—		—		*bla*_CMY_*, bla*_IMP_	
Cefpirome (4th CEP)	32	0.25		0.25		—			—		—		*bla*_CMY_*, bla*_IMP_	
Cefepime (4th CEP)	32	0.25		0.25		—			—		—		*bla*_CMY_*, bla*_IMP_	
Aztreonam (MONO)	8	0.13		0.25		—			—		—		*bla*_CMY_*, bla*_IMP_	
Imipenem (CARB)	16	0.5		0.5		—			—		—		*bla*_IMP_	
Meropenem (CARB)	32	≤0.03		≤0.03		—			—		—		*bla*_IMP_	
Aminoglycosides														
Streptomycin	>128	4		>128 ^b^		*acc(6)-Id*			1693		1504		*aadA*	
Tobramycin	128	32		32		*aac(6′)-Ic*			3758		3638		*aadA*	
Amikacin	64	16		16		*aac(6′)-Ic*			3758		3638		*aadA*	
Arbekacin	64	16		16		*aac(6′)-Ic*			3758		3638		*aadA*	
Kanamycin	128	32		32		*aac(6′)-Ic*			3758		3638		*aadA*	
Spectinomycin	>512	32		16		*aac(6′)-Ic*			3758		3638		*aadA*	
Gentamycin	16	4		4		—			—		—		*aadA*	
Quinolones														
Levofloxacin	4	4		0.25		*gyrA*^c^			[+] in 2886		[−] in 2665		—	
Sparfloxacin	4	4		0.5		*gyrA*^c^			[+] in 2886		[−] in 2665		—	
Ciprofloxacin	4	4		0.25		*gyrA*^c^			[+] in 2886		[−] in 2665		—	
Tosufloxacin	2	2		0.125		*gyrA*^c^			[+] in 2886		[−] in 2665		—	
Macrolides														
Erythromycin	128	128		128		*macAB*			1134_1135		0964_0965		—	
Josamycin	>512	>512		>512		*macAB*			1134_1135		0964_0965		—	
Others														
Tetracycline	16	16		64		*tetA*			Not found		1759		—	
Rifampicin	16	16		16		—			—		—		—	
Fosfomycin	>512	>512		>512		*fosA*			2306		2100		—	
Sulfamethoxazole	>128	>128		>128					Unknown		Unknown		*sulI1/2*	
Ethidium bromide	>512	>512		>512		*ssmE*			2035		1855		*qacE1*/*2*	
Benzalkonium chloride	128	128		128		—			—		—		—	
HgCl_2_	32	8		8		—			—		—		*mer* operon	

Note.—PEN, penicillin; PEM, penem; 1st CEP, 1st generation cephalospoin; 2nd CEP, 2nd generation cephalospoin; 3rd CEP, 3rd generation cephalospoin; 4th CEP, 4th generation cephalospoin; MONO, monobactam; CARB, carbapenem.

^a^KS3 is a pSMC1-cured SM39 derivative.

^b^The *rpsL* gene of Db11 contains a point mutation which confers streptomycin resistance on this strain.

^c^Mutation in *gyrA*. The S83R mutation in the quinolone resistance-determining region.

#### Acquired Antimicrobial Resistance Genes

**Fig. 6.—:**
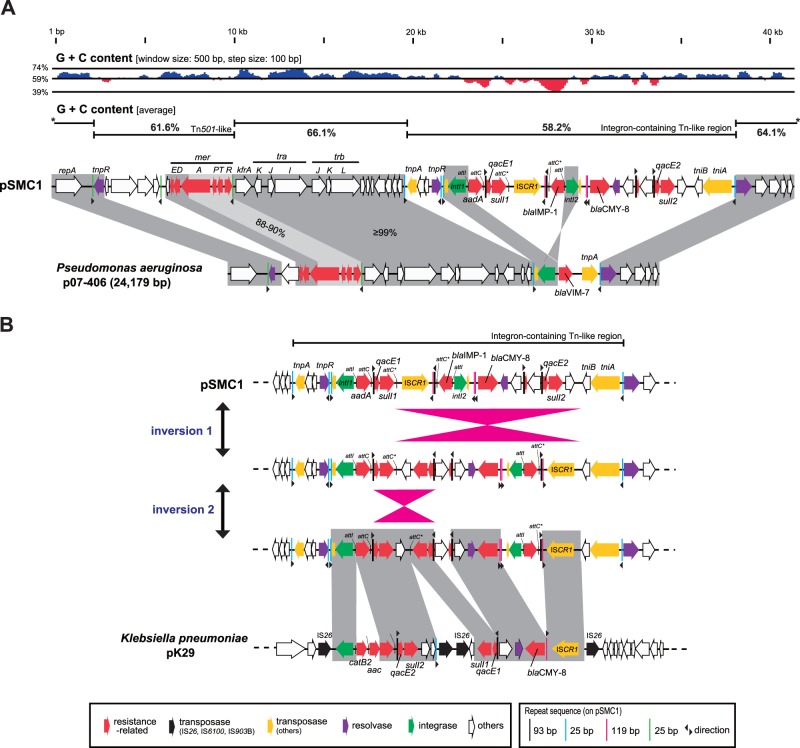
Genomic features and a possible evolutionary process of the pSMC1 multidrug-resistant plasmid of SM39. (*A*) Genomic comparison of pSMC1 and p07-406, an IncP plasmid of *Pseudomonas aeruginosa*. Although significant differences were observed in the region corresponding to a Tn*501*-like transposon and the region corresponding to an integron-carrying transposon, the backbones of the two plasmid genomes are nearly identical and their GC content is significantly higher than that of the *Serratia marcescens* chromosomes and rather similar to that of *Pseudomonas* species, suggesting that pSMC1 originated from *Pseudomonas* species. (*B*) Structural comparison of the integron of pSMC1 with that on pK29 of *Klebsiella pneumoniae*. The difference in genetic structure between the two integrons could be generated by two inversion events and by insertion of IS elements and acquisition/duplication of several genes.

Unlike Db11, SM39 carries two plasmids. The overall GC contents of the two plasmids differ significantly from the chromosome (∼59%): 61.5% for pSMC1 and 51.9% for pSMC2. The plasmid pSMC2 carries a set of genes for conjugation (the *tra* and *trb* genes), and although lacking *traJ* and *traT*, it can be transferred by conjugation from SM39 to *E. coli* K-12 strain x1037 Rif^r^ (data not shown). Although pSMC2 contains no gene related to virulence or drug resistance, pSMC1 carries an integron that contains the *aadA* gene encoding an aminoglycoside-3′-adenylyltransferase, the *bla*CMY-8 gene encoding a class C beta-lactamase which inactivates a much broader range of beta-lactams than the chromosomally encoded class C beta-lactamase (AmpC), and the *bla*IMP-1 gene encoding a metal beta-lactamase (MBL). In addition, pSMC1 contains two copies of the *qacE* gene, associated with resistance to a range of disinfectants (Kucken et al. 2000), and two copies of the sulfonamide resistance gene (*sulI*) as well as a transposon encoding the *mer* operon for mercury resistance (fig. 6*A*).

Comparison of a pSMC1-cured SM39 derivative (KS3) with the wild type showed that the high level of resistance displayed by SM39 to beta-lactams, aminoglycosides and mercury is accounted for by the presence of pSMC1. Compared with SM39, KS3 was great or equal to four more sensitive to 22 of the 36 antimicrobial agents tested; 14 of the 16 beta-lactams, all of the seven aminoglycosides, and to mercury. The 14 beta-lactams included carbapenems, the resistance to which is attributable to MBL. The exception is resistance to fluoroquinolones, which, as mentioned above, is attributable to the chromosomal mutation in *gyrA* (table 3).

Plasmid pSMC1 exhibits a remarkable similarity to an IncP plasmid of *Pseudomonas aeruginosa,* p07-406 (Li et al. 2008) (fig. 6*A*). The backbones of the two plasmids encoding the *tra* region and replication functions are nearly identical in gene compliment and with a GC content (64.1–66.1%) characteristic of pseudomonads. The main differences concern their genetic cargo encoding multiple drug resistance. This is consistent with the notion that *Pseudomonas* species are the original hosts for IncP plasmids with high GC backbones (Thorsted et al. 1998). However, as the integron-containing region of pSMC1 is also high similarity to that on plasmid pK29 of *Klebsiella pneumoniae* (Chen et al. 2007) although complex rearrangements including inversion and gene duplication have occurred, it suggests that these closely related plasmids have become widely disseminated in the *Enterobacteriaceae* (fig. 6*B*).

## Concluding Remarks

We selected two *S. marcescens* strains from contrasting sources in an attempt to maximize our ability to capture the genomic diversity of this ubiquitous enteric species. Through our comparative analysis, we have defined a core genome of *S. marcescens* as well as that of the genus, although it should be noted that the latter gene set was generated from free-living *Serratia* species only. The former gene set defines the intrinsic metabolic capacities, virulence, and multidrug resistance of this opportunistic pathogen. Analysis of strain-specific genes or genomic regions has further revealed a high level of genomic diversity and plasticity of *S. marcescens*, which reflects the diversity of niches that this species can occupy. In fact, among the SM39-specific genes, many genes that are implicated in the high virulence potential of this clinical isolate have been identified, this includes many virulence-related genes found within the core genome of *S. marcescens*. Furthermore, analysis of the pSMC1 plasmid of SM39 has revealed that it encodes MBL and many other drug resistance determinants and is responsible for the extremely high level of multidrug resistance of the strain. We were able to propose a possible origin and evolution of pSMC1 on the basis of its genomic features. These data, combined with available tools for functional genomic analysis such as that described by Pettyet al. (2006: 1701–1708), will accelerate research on *S. marcescens* in numerous domains and provide new insights into the genetic mechanisms responsible for the emergence of pathogens highly resistant to multiple antimicrobial agents.

## Supplementary Material

Supplementary figures S1–S5 and tables S1–S9 are available at *Genome Biology and Evolution* online (http://www.gbe.oxfordjournals.org/).

Supplementary Data
