# Supplementary material for: Genome Evolution and Plasticity of Serratia marcescens, an Important Multidrug-Resistant Nosocomial Pathogen
Source: Genome Biol Evol. 2014 Jul 28;6(8):2096–110. doi: 10.1093/gbe/evu160 (PMC4231636; doi:10.1093/gbe/evu160)
Supplement: Supplementary Data [file supp_6_8_2096__index.html]

Genome Evolution and Plasticity of Serratia marcescens, an Important Multidrug-Resistant Nosocomial Pathogen — Supplementary Data 

# Genome Evolution and Plasticity of *Serratia marcescens*, an Important Multidrug-Resistant Nosocomial Pathogen

## Supplementary Data

files

**Files in this Data Supplement:**

- Supplementary Data - pdf file
- Supplementary Data - pdf file
