# Supplementary material for: Genome Evolution and Plasticity of Serratia marcescens, an Important Multidrug-Resistant Nosocomial Pathogen
Source: Genome Biol Evol. 2014 Jul 28;6(8):2096–110. doi: 10.1093/gbe/evu160 (PMC4231636; doi:10.1093/gbe/evu160)
Supplement: Supplementary Data [file supp_evu160_SMA_20140703_SuppleFigs_igu2.pdf]

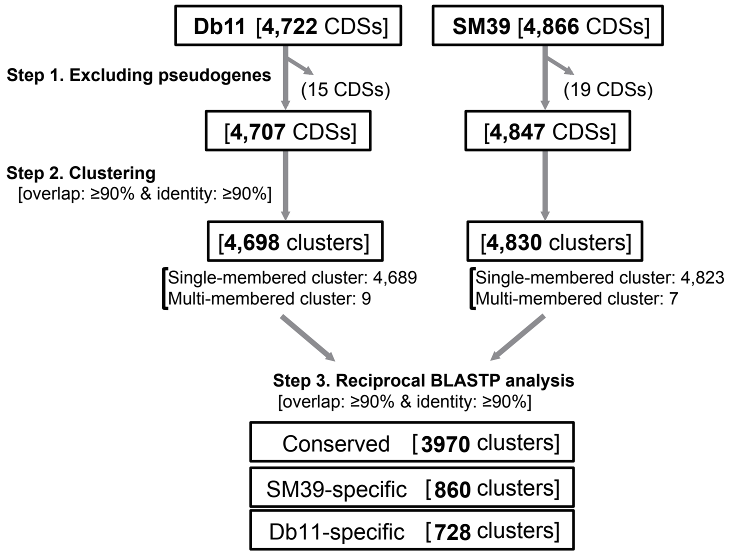
Db11 [4,722 CDSs]
SM39 [4,866 CDSs]
Step 1. Excluding pseudogenes
(15 CDSs)
(19 CDSs)
[4,707 CDSs]
[4,847 CDSs]
Step 2. Clustering
[overlap: ≥90% & identity: ≥90%]
[4,698 clusters]
[4,830 clusters]
Single-membered cluster: 4,689
Multi-membered cluster: 9
Single-membered cluster: 4,823
Multi-membered cluster: 7
Step 3. Reciprocal BLASTP analysis
[overlap: ≥90% & identity: ≥90%]
Conserved [3970 clusters]
SM39-specific [860 clusters]
Db11-specific [728 clusters]

Figure S1

A. Construction of pPAR002

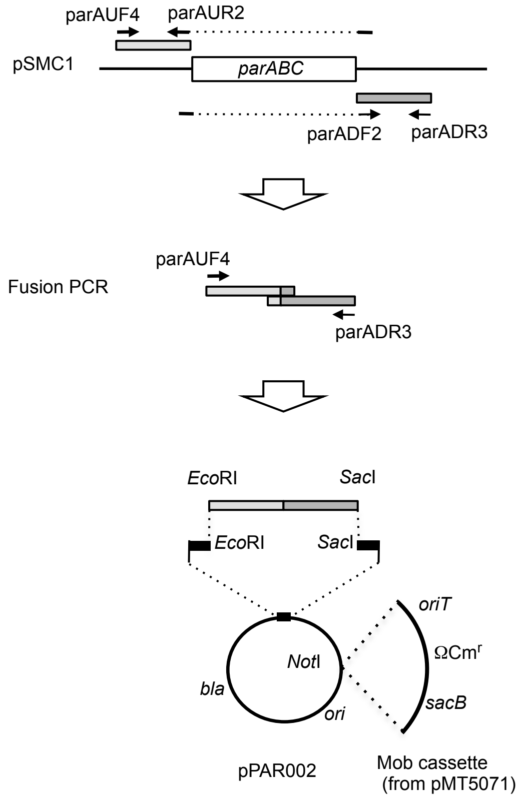

B. Curing of pSMC1 from SM39

1. Transformation of pPAR002 into *E. coli* S17-1
2. Conjugal transfer of pPAR002 into SM39 on LB plates
3. Select trans-conjugants (SM39 carrying pPAR002) on BM2-Cm (150 μg/ml) plates*
4. 1st recombination event:
   Screening of sucrose^s colonies on LB and LB-10% sucrose plates
   Confirmation of Cm^r on BM2-Cm (150 μg/ml) plates

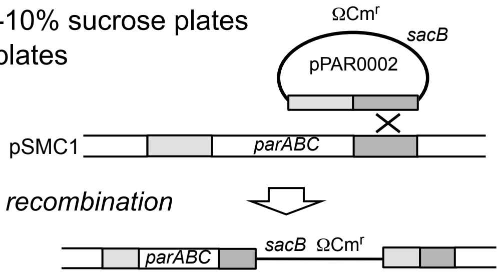

1st homologous recombination

   Confirmation of the 1st recombination by PCR

5. 2nd recombination event:
   Screening sucrose^r colonies on LB-10% sucrose plates

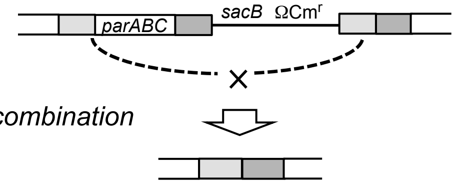

2nd homologous recombination

Confirmation of the 2nd recombination by PCR
and lack of growth on LB-Cm (150 μg/ml) plates

pSMC1-cured derivatives were confirmed by the loss of $HgCl_2$-resistance on LB-$HgCl_2$ (16 μg/ml) plates

*BM2 medium: 0.7 % K_2HPO_4, 0.3% KH_2PO_4, 0.1 % $(NH_4)_2SO_4$, 0.01 % $MgSO_4 \cdot 7H_2O$, 1.08 % sodium succinate, 1.5 % agarose

Figure S2

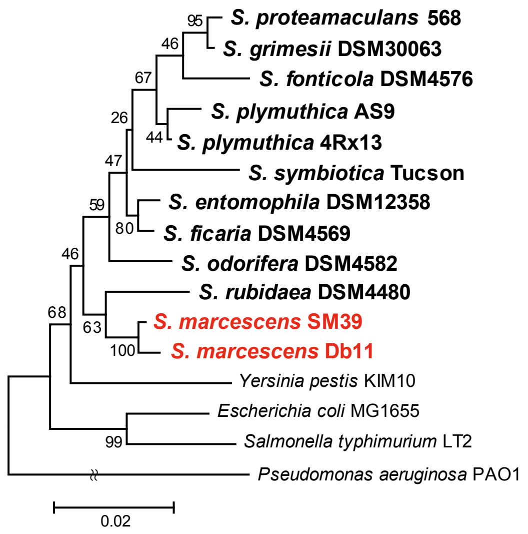
S. proteamaculans 568
S. grimesii DSM30063
S. fonticola DSM4576
S. plymuthica AS9
S. plymuthica 4Rx13
S. symbiotica Tucson
S. entomophila DSM12358
S. ficaria DSM4569
S. odorifera DSM4582
S. rubidaea DSM4480
S. marcescens SM39
S. marcescens Db11
Yersinia pestis KIM10
Escherichia coli MG1655
Salmonella typhimurium LT2
Pseudomonas aeruginosa PAO1
95
46
67
26
44
47
59
80
46
68
63
100
99
0.02

Figure S3

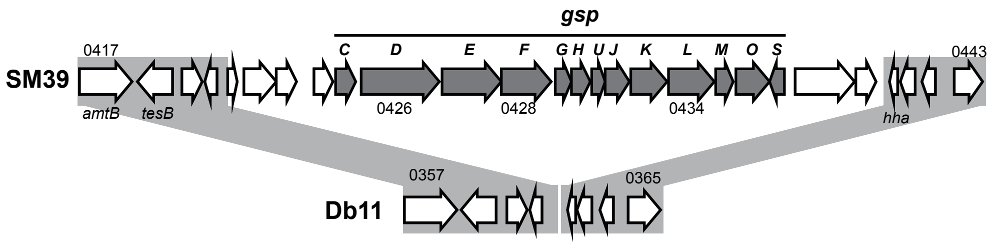
gsp
C D E F G H U J K L M O S
SM39
0417
0443
amtB
tesB
0426
0428
0434
hha
Db11
0357
0365

Figure S4

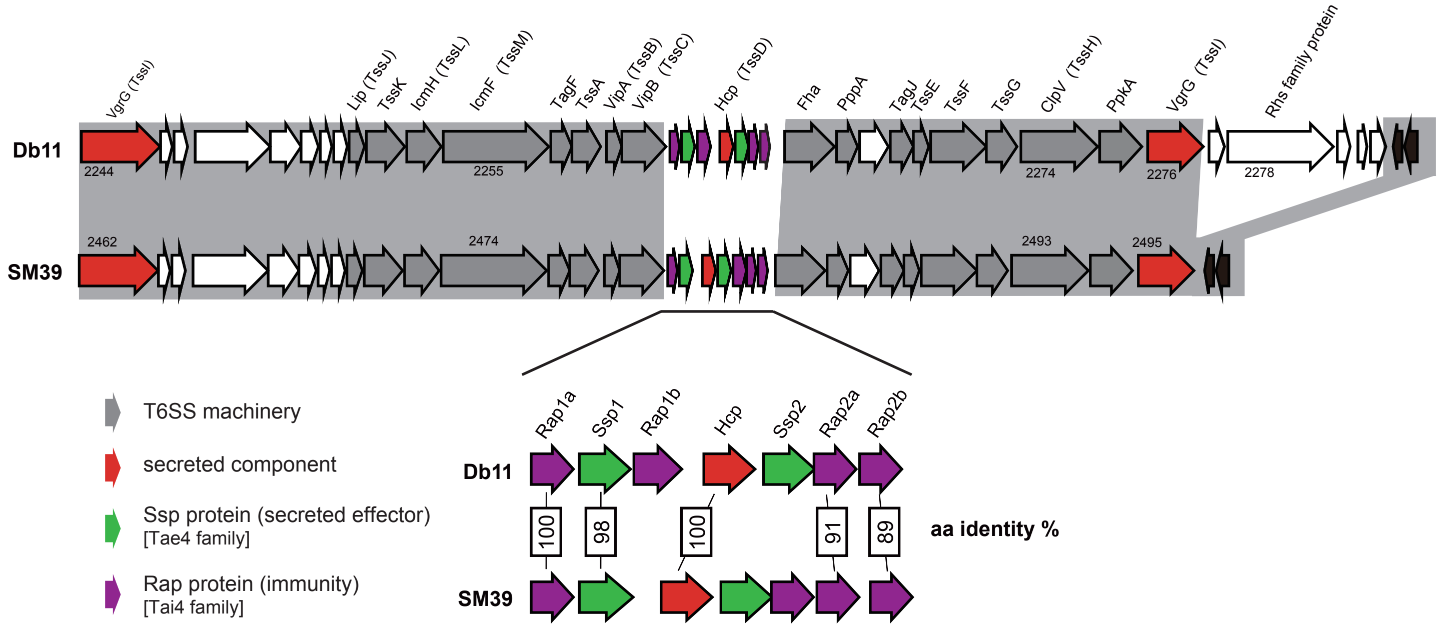

Db11
SM39
VgrG (TssI)
Lip (TssJ)
TssK
IcmH (TssL)
IcmF (TssM)
TagF
TssA
VipA (TssB)
VipB (TssC)
Hcp (TssD)
Fha
PppA
TagJ
TssE
TssF
TssG
ClpV (TssH)
PpkA
VgrG (TssI)
Rhs family protein
2244
2255
2274
2276
2278
2462
2474
2493
2495
T6SS machinery
secreted component
Ssp protein (secreted effector)
[Tae4 family]
Rap protein (immunity)
[Tai4 family]
Rap1a
Ssp1
Rap1b
Hcp
Ssp2
Rap2a
Rap2b
100
98
100
91
89
aa identity %

Figure S5
