# Supplementary material for: Genome Evolution and Plasticity of Serratia marcescens, an Important Multidrug-Resistant Nosocomial Pathogen
Source: Genome Biol Evol. 2014 Jul 28;6(8):2096–110. doi: 10.1093/gbe/evu160 (PMC4231636; doi:10.1093/gbe/evu160)
Supplement: Supplementary Data [file supp_evu160_SMA_20140717_SuppleTables_igu2.pdf]

Table S1. SM39-specific regions that are absent in Db11 (sequence identity: ≤90%, length: ≥5 kb)

Elements	Start	End	Size (bp)	Genes	
SM39_E01	226552	267390	40839	SM39_0197-0244	SM39_PP1 (Mu-like)
SM39_E02	295636	307714	12079	SM39_0274-0284	two-component system
SM39_E03	446028	461861	15834	SM39_0421-0439	T2SS
SM39_E04	494011	503704	9694	SM39_0471-0476	NRPS
SM39_E05	511606	530321	18716	SM39_0484-0499	allantoin utilization (*all-glx* gene set)
SM39_E06	545665	598458	52794	SM39_0516-0594	SM39_PP2 (lambda-like)
SM39_E07	756427	761881	5455	SM39_0746-0749	
SM39_E08	945910	954763	8854	SM39_0942-0948	
SM39_E09	1030487	1050917	20431	SM39_1016-1031	exopolysaccharide (capsule)
SM39_E10	1060270	1071046	10777	SM39_1037-1045	exopolysaccharide (O-antigen)
SM39_E11	1118420	1124755	6336	SM39_1092-1098	
SM39_E12	1262274	1297061	34788	SM39_1219-1265	SM39_PP3
SM39_E13	1299328	1309287	9960	SM39_1270-1280	fimbrial locus 3
SM39_E14	1456828	1461949	5122	SM39_1436-1439	
SM39_E15	1464099	1474246	10148	SM39_1443-1448	beta-galactosidase (*ebg* operon)
SM39_E16	1810086	1815741	5656	SM39_1778-1782	microcin
SM39_E17	1878006	1889041	11036	SM39_1844-1855	serralysin-like protease
SM39_E18	1911520	1924983	13464	SM39_1877-1887	nitrate reductase (*nirBDC-cysG*)
SM39_E19	1952952	1964577	11626	SM39_1912-1918	pump (RND)
SM39_E20	1987305	1993333	6029	SM39_1936-1940	
SM39_E21	1995876	2005506	9631	SM39_1946-1953	
SM39_E22	2138241	2152393	14153	SM39_2077-2080	Cdi system
SM39_E23	2185446	2199489	14044	SM39_2069-2130	SM39_PP4
SM39_E24	2628862	2639496	10635	SM39_2581	NRPS
SM39_E25	2644633	2654233	9601	SM39_2586-2597	fimbrial locus 6
SM39_E26	2657088	2674098	17011	SM39_2602-2615	
SM39_E27	2693415	2700533	7119	SM39_2637-2643	
SM39_E28	2720328	2749203	28876	SM39_2665-2696	SM39_IE1, type III RM system, plasmid-related genes
SM39_E29	2900511	2918186	17676	SM39_2837-2855	SM39_PP5 (lambda-like)
SM39_E30	2949991	2959037	9047	SM39_2882	glycosyltransferase
SM39_E31	3075958	3084814	8857	SM39_3003-3008	haemin storage system (*hms* operon)
SM39_E32	3104380	3112591	8212	SM39_3028-3034	fimbrial locus 7
SM39_E33	3218185	3230644	12460	SM39_3139-3145	Cdi system
SM39_E34	3390342	3396587	6246	SM39_3295-3300	
SM39_E35	3755005	3767973	12969	SM39_3638-3650	fimbrial locus 8
SM39_E36	3854719	3878288	23570	SM39_3729-3758	SM39_PP6, type I RM system
SM39_E37	4002004	4019806	17803	SM39_3884	NRPS (*swrA*-like)
SM39_E38	4227980	4234716	6737	SM39_4088-4095	L-ascorbate utilization (*ula* operon)
SM39_E39	4254093	4263160	9068	SM39_4110-4118	
SM39_E40	4281738	4289275	7538	SM39_4138-4145	fimbrial locus 9
SM39_E41	4768046	4795457	27412	SM39_4574-4613	SM39_PP7 (P2-like)
SM39_E42	5047976	5070272	22297	SM39_4836-4858	SM39_IE2, LuxI/R family, type I RM system
SM39_E43	5071502	5078868	7367	SM39_4862-4873	
SM39_E44	5089692	5099882	10191	SM39_4889-4896	

Table S2. Db11-specific regions that are absent in SM39 (sequence identity: ≤90%, length: ≥5 kb)

Element	Start	End	Size (bp)	Genes	
Db11_E01	635131	642854	7724	SMDB11_0601-0606	bacteriocin
Db11_E02	883180	900361	17182	SMDB11_0849-0863	exopolysaccharide (capsule)
Db11_E03	909728	923722	13995	SMDB11_0870-0878	exopolysaccharide (O-antigen)
Db11_E04	1116229	1123942	7714	SMDB11_1052-1058	fimbrial locus 3
Db11_E05	1165437	1175965	10529	SMDB11_101-1108	DB11_IE1
Db11_E06	1310880	1318663	7784	SMDB11_1251-1257	pump (RND)
Db11_E07	1360574	1365622	5049	SMDB11_1296-1299	
Db11_E08	1376716	1381973	5258	SMDB11_1311-1319	
Db11_E09	1675673	1682711	7039	SMDB11_1598-1601	type IR/M (*hsd* operon)
Db11_E10	1693465	1700954	7490	SMDB11_1610-1621	Rhs
Db11_E11	1718118	1724027	5910	SMDB11_1640-1645	ABC-transporter
Db11_E12	1734702	1747939	13238	SMDB11_1658-1663	Rhs
Db11_E13	1788581	1794001	5421	SMDB11_1697-1700	pump (RND)
Db11_E14	1795619	1801415	5797	SMDB11_1702-1710	
Db11_E15	1924070	1933713	9644	SMDB11_1815-1823	
Db11_E16	2232481	2238239	5759	SMDB11_2122-2126	
Db11_E17	2402661	2409472	6812	SMDB11_2277-2281	Rhs
Db11_E18	2414889	2442511	27623	SMDB11_2288-2293	NRPS (*alb1*-*alb6*)
Db11_E19	2455828	2461047	5220	SMDB11_2303A-2310	
Db11_E20	2543898	2552130	8233	SMDB11_2400-2408	DB11_IE3
Db11_E21	2556518	2588505	31988	SMDB11_2416-2443	
Db11_E22	2748342	2787893	39552	SMDB11_2587-2638	DB11_PP2 (Mu-like)
Db11_E23	3037072	3043902	6831	SMDB11_2878-2884	
Db11_E24	3122793	3130168	7376	SMDB11_2956-2961	
Db11_E25	3252355	3261427	9073	SMDB11_3079-3083	
Db11_E26	3418295	3423461	5167	SM3224-3226A	fimbrial locus 6
Db11_E27	3438262	3444382	6121	SMDB11_3241-3244	
Db11_E28	3465167	3474140	8974	SMDB11_3265-3271	
Db11_E29	3524742	3537481	12740	SMDB11_3326-3337	*sgb* operon (L-ascorbate utilization)
Db11_E30	3583779	3592155	8377	SMDB11_3379-3385	fimbrial locus 7
Db11_E31	3759689	3765569	5881	SMDB11_3533-3540	
Db11_E32	3859441	3875909	16469	SMDB11_3637-3654	
Db11_E33	3903165	3920880	17716	SMDB11_3680	NRPS (*swrA*)
Db11_E34	4165237	4174115	8879	SMDB11_3913-3921	fimbrial locus 8
Db11_E35	4182201	4188451	6251	SMDB11_3927-3933	
Db11_E36	4502272	4521338	19067	SMDB11_4212-4228	bacteriocin-related genes, two component system
Db11_E37	4762911	4789989	27079	SMDB11_4418-4449	fimbrial locus 9 and locus 10
Db11_E38	4899105	4918801	19697	SMDB11_4555-4572	plasmid-related genes
Db11_E39	4985507	4991922	6416	SMDB11_4637-4641	exopolysaccharide

Table S3. IS elements identified in SM39 and Db11

IS element	SM39	Db11	Location	Most similar IS (% DNA sequence idenitty) [a]	Target site duplication (bp) [b]	Terminal inverted repeat (bp)
IS*Se1*[c] (IS*3* family)	6		chromosome	-	3	24
IS*Se2*[c] (IS*4* family)	5		chromosome	-	10-12	21
IS*Se3*[c] (IS*3* family)		2	chromosome	-	ND	17
IS*10L* (IS*4* family)	1		chromosome	-	9	22
IS*Se4*[c] (IS*3* family)	1		chromosome	IS*Ec31* (87%)	ND	24
IS*Se5*[c] (IS*3* family)	1[d]	1[d]	chromosome	IS*Ec14* (92%)	ND	26
IS*Se6*[c] (IS*256* family)	1		chromosome	IS*1414* (85%)	8	30
IS*Se7*[c] (IS*66* family)	1		chromosome	IS*Ec22* (87%)	8	22
IS*Se8*[c] (IS*4* family)		1	chromosome	IS*Cro6* (88%)	ND	19/20
IS*Se9*[c] (IS*3* family)		1	chromosome	IS*Raq1* (93%)	ND	21/22
IS*CR1* (IS*91*-like)	1		pSMC1	-	ND	ND
total	17	5				

[a] -; the closest IS shows less than 70% nucleotide sequence identity.

[b] ND; not defined.

[c] These IS elements were newly identified in this study.

[d] These two IS elements show 96.7% nucleotide sequence identity.

Table S4. Prophages and integrative elements of SM39 and Db11

Name	Length (bp)	Start position	End position	Insertion site	type [a]	Direct repeat (bp)	Features
SM39							
SM39_PP1	38404	226473	264876	SM39_0196/SM39_0243	PP	ND	Mu-like phage
SM39_PP2	45019	545889	590907	*arg* (tRNA)	PP	47	lambda-like phage
SM39_PP3	34800	1262262	1297061	SM39_1218/SM39_1266	PP	12	
SM39_PP4	14044	2185446	2199489	*rluB*/Se2131	PP	16	
SM39_PP5	17245	2900955	2918199	*thr* (tRNA)	PP	11	lambda-like phage (defective), integrase (-)
SM39_PP6	22292	3855402	3877693	*met* (tRNA)	PP	29	type I RM system
SM39_PP7	27459	4767999	4795457	*cpxP_fieF*	PP	30	P2-like phage
SM39_IE1	28901	2720303	2749203	*asn* (tRNA)	IE	28	type III RM system, plasmid-related genes
SM39_IE2	10865	5059408	5070272	*leu* (tRNA)	IE	ND	type I RM system
Db11							
DB11_PP1	4172	1238874	1243045	*ptsG/SMDB11_1178*	PP	ND	P2-like (partial)
DB11_PP2	39599	2748295	2787893	*pro* (tRNA)	PP	47	Mu-like
DB11_IE1	6168	1165432	1171599	*gly* (tRNA)	IE	ND	
DB11_IE2	2312	1278868	1281179	SMDB11_1216/SMDB11_1219	IE	ND	
DB11_IE3	8255	2543876	2552130	*asn* (tRNA)	IE	22	
DB11_IE4	1380	4975064	4976444	*leu* (tRNA)	IE	ND	

[a] PP; prophages, IE; integrative elements that encode an integrase but not other phage-related functions.

Table S5. Secreted or surface-exposed proteins of known or predictable functions

	SM39	Db11	Gene	Product	Amino-acid sequence identity between SM39 and Db11	Prevalence of homologues in other *Serratia* strains			
						S. plymuthica AS9	*S. plymuhica* 4Rx13	*S. odorifera* DSM4582	*S. proteamaculans* 568
**Predictable signal peptide (+)**									
	SM39_0386	-	-	hemagglutinin-like protein	-	-	-	-	-
	SM39_1283	SMDB11_1061	-	putative nuclease	100.0%	+	+	+	+
	-	SMDB11_1670	-	extracellular serine protease (with autotransporter domain)	-	-	-	-	-
	SM39_2024	SMDB11_1844	-	putative secreted serine protease	97.8%	+	+	+	+
	SM39_2079	-	-	CdiA-family protein	-	-	-	+	+
	SM39_2198	SMDB11_1994	-	chitinase	96.7%	+	+	-	+
	SM39_2810	SMDB11_2573	*spr*	predicted peptidase, outer membrane lipoprotein	99.0%	+	+	+	+
	SM39_2958	SMDB11_2756	-	endonuclease/exonuclease/phosphatase familyprotein	98.3%	+	+	-	+
	SM39_3079	SMDB11_2863	-	serine protease	98.6%	-	-	-	+
	SM39_3125	SMDB11_2916	-	putative exported peptidase	99.2%	+	+	+	+
	SM39_3144	-	-	CdiA-family protein	-	-	+	-	-
	SM39_3279	SMDB11_3069	*phlA*	extracellular phospholipase A1	98.1%	+	+	-	+
	SM39_3936	SMDB11_3737	*shlA*	hemolysin	95.3%	+	+	-	+
	SM39_4178	SMDB11_3961	*ssph2*	extracellular serine protease SSP-h2 (with autotransporter domain)	96.8%	+	+	-	+
	SM39_4309	SMDB11_4295	*pldA*	outer membrane phospholipase A	98.6%	+	+	+	+
	SM39_4347	SMDB11_4259	-	putative exported zinc-protease	98.8%	+	+	+	+
	SM39_4365	SMDB11_4243	*chiA*	endo-chitinase	98.9%	+	+	+	+
	SM39_4420	SMDB11_4176	*estA*	lipase/esterase (with autotransporter domain)	97.7%	+	+	+	+
	SM39_4935	SMDB11_4679	-	putative phospholipase	97.6%	+	+	+	+
	SM39_3091	SMDB11_2877	*cbp*	chitin binding protein	98.5%	+	+	+	+
**Predictable signal peptide (-)**[a]									
	SM39_0641	SMDB11_0468	*chiC*	chitinase	95.8%	+	+	+	+
	SM39_1031	SMDB11_0863	*slaA*	surface layer protein	77.2%	-	-	-	-
	-	SMDB11_1367		extracellular metalloproteinase (serralysin family)	-	-	-	-	-
	SM39_1673	SMDB11_1483	-	extracellular lipase	96.6%	+	+	+	+
	SM39_1793	SMDB11_1606	-	extracellular metalloproteinase (serralysin family)	99.3%	-	-	-	-
	SM39_1845	-	-	extracellular metalloproteinase (serralysin family)	-	-	+	-	+
	SM39_2716	SMDB11_2486	-	extracellular metalloproteinase (serralysin family)	92.8%	-	-	-	-
	SM39_3089	SMDB11_2875	*chiB*	chitinase	98.4%	+	+	-	+
	SM39_3192	SMDB11_2982	-	autotransporter domain-containing protein	96.9%	+	+	+	+
	SM39_4179	SMDB11_3962	*ssph1*	extracellular serine protease SSP-h1 (with autotransporter domain)	95.8%	+	+	+	+
	SM39_4293	SMDB11_4311	-	serralysin precursor	99.4%	+	+	+	+
**Type VI secretion system-associated**									
	SM39_2481	SMDB11_2263	*hcp1*	Hcp homologue	100.0%	-	-	+	+
	-	SMDB11_3455	*hcp2*	Hcp homologue	-	-	-	+	+
	-	SMDB11_3456	*hcp3*	Hcp homologue	-	-	-	-	-
	SM39_2462	SMDB11_2244	*vgrG1*	VgrG homologue	96.8%	-	-	-	-
	SM39_2495	SMDB11_2276	*vgrG2*	VgrG homologue	98.9%	-	-	+	+
	SM39_2480	SMDB11_2261	*ssp1*	Type VI secreted amidase effector	98.2%	-	-	-	-
	-	SMDB11_2264	*ssp2*	Type VI secreted amidase effector[b]	-	-	-	-	-
	SM39_2482	-		Putative secreted amidase effector[b]	-	-	-	-	-

[a] Although the presence of signal peptides were not predicted for these gene products by the SignalP program, their localizations (surface-exposed or secreted) were predicted from the experimental data of the homologs in other S. marcescens strains. For example, SSP-H1 has been shown to be localized in outer membrane (Ohnishi Y. et al., J. Biochem., 121(5): 902-913, 1996).

[b] SMDB11_2264 shows 42.0% amino-acid sequence identity to SM39_2482

Table S6. Flagellar biosynthesis- and chemotaxis-related genes in the genomes of SM39 and Db11

function	locus tag (*E. coli* K-12)	gene	product	orthologs in *Serratia marcescens*		amino acid sequcnce identity between SM39 and Db11
				SM39	Db11	
flagellar biosynthesis	b1070	*flgN*	export chaperone for FlgK and FlgL	SM39_2435	SMDB11_2222	98.6
	b1071	*flgM*	anti-sigma factor for FliA (sigma 28)	SM39_2434	SMDB11_2221	97.1
	b1072	*flgA*	assembly protein for flagellar basal-body periplasmic P ring	SM39_2433	SMDB11_2220	96.8
	b1073	*flgB*	flagellar component of cell-proximal portion of basal-body rod	SM39_2432	SMDB11_2219	97.8
	b1074	*flgC*	flagellar component of cell-proximal portion of basal-body rod	SM39_2431	SMDB11_2218	100
	b1075	*flgD*	flagellar hook assembly protein	SM39_2430	SMDB11_2217	99.1
	b1076	*flgE*	flagellar hook protein	SM39_2429	SMDB11_2216	96.8
	b1077	*flgF*	flagellar component of cell-proximal portion of basal-body rod	SM39_2428	SMDB11_2215	99.6
	b1078	*flgG*	flagellar component of cell-distal portion of basal-body rod	SM39_2427	SMDB11_2214	100
	b1079	*flgH*	flagellar protein of basal-body outer-membrane L ring	SM39_2426	SMDB11_2213	100
	b1080	*flgI*	predicted flagellar basal body protein	SM39_2425	SMDB11_2212	100
	b1081	*flgJ*	muramidase	SM39_2424	SMDB11_2211	99
	b1082	*flgK*	flagellar hook-filament junction protein 1	SM39_2423	SMDB11_2209	99.1
	b1083	*flgL*	flagellar hook-filament junction protein	SM39_2422	SMDB11_2208	98.8
	b1879	*flhA*	predicted flagellar export pore protein	SM39_2441	SMDB11_2225	99.4
	b1880	*flhB*	predicted flagellar export pore protein	SM39_2442	SMDB11_2226	99.5
	b1889	*motB*	protein that enables flagellar motor rotation	SM39_2453	SMDB11_2235	94.1
	b1890	*motA*	proton conductor component of flagella motor	SM39_2454	SMDB11_2236	100
	b1891	*flhC*	DNA-binding transcriptional dual regulator with FlhD	SM39_2455	SMDB11_2237	100
	b1892	*flhD*	DNA-binding transcriptional dual regulator with FlhC	SM39_2456	SMDB11_2238	99.1
	b1920	*fliY*	cystine transporter subunit	SM39_2392	SMDB11_2182	97.4
	b1921	*fliZ*	predicted regulator of FliA activity	SM39_2394	SMDB11_2184	98.2
	b1922	*fliA*	RNA polymerase, sigma 28 (sigma F) factor	SM39_2395	SMDB11_2185	100
	b1923	*fliC*	flagellar filament structural protein (flagellin)	SM39_2396	SMDB11_2186	68.3
	b1924	*fliD*	flagellar filament capping protein	SM39_2397	SMDB11_2187	78
	b1925	*fliS*	flagellar protein potentiates polymerization	SM39_2398	SMDB11_2188	97.8
	b1926	*fliT*	predicted chaperone	SM39_2399	SMDB11_2189	100
	b1937	*fliE*	flagellar basal-body component	SM39_2405	SMDB11_2194	98.1
	b1938	*fliF*	flagellar basal-body MS-ring and collar protein	SM39_2406	SMDB11_2195	98.2
	b1939	*fliG*	flagellar motor switching and energizing component	SM39_2407	SMDB11_2196	99.4
	b1940	*fliH*	flagellar biosynthesis protein	SM39_2408	SMDB11_2197	97
	b1941	*fliI*	flagellum-specific ATP synthase	SM39_2409	SMDB11_2198	99.8
	b1942	*fliJ*	flagellar protein	SM39_2410	SMDB11_2199	98
	b1943	*fliK*	flagellar hook-length control protein	SM39_2411	SMDB11_2200	90.2
	b1944	*fliL*	flagellar biosynthesis protein	SM39_2412	SMDB11_2201	98.1
	b1945	*fliM*	flagellar motor switching and energizing component	SM39_2413	SMDB11_2202	99.7
	b1946	*fliN*	flagellar motor switching and energizing component	SM39_2414	SMDB11_2203	98.6
	b1947	*fliO*	flagellar biosynthesis protein	SM39_2415	SMDB11_2204	98.5
	b1948	*fliP*	flagellar biosynthesis protein	SM39_2416	SMDB11_2205	99.6
	b1949	*fliQ*	flagellar biosynthesis protein	SM39_2417	SMDB11_2206	100
	b1950	*fliR*	flagellar export pore protein	SM39_2418	SMDB11_2207	98.5
chemotaxis-related	b1881	*cheZ*	chemotaxis regulator, protein phosphatase for CheY	SM39_2445	SMDB11_2227	100
	b1882	*cheY*	chemotaxis regulator transmitting signal to flagellar motor component	SM39_2446	SMDB11_2228	99.2
	b1883	*cheB*	fused chemotaxis regulator: protein-glutamate methylesterase in two-component regulatory system with CheA	SM39_2447	SMDB11_2229	99.1
	b1884	*cheR*	chemotaxis regulator, protein-glutamate methyltransferase	SM39_2448	SMDB11_2230	100
	b1887	*cheW*	purine-binding chemotaxis protein	SM39_2451	SMDB11_2233	100
	b1888	*cheA*	fused chemotactic sensory histidine kinase in two-component regulatory system with CheB and CheY: sensory histidine kinase/signal sensing protein	SM39_2452	SMDB11_2234	95.4
methyl-accepting chemotaxis proteins	na	na	methyl-accepting chemotaxis protein (PAS*1 & MCP domains)	SM39_0916	SMDB11_0747	98.4
	na	na	methyl-accepting chemotaxis protein (TarH*2, HAMP*3 & MCP domains)	SM39_1816	SMDB11_1636	91.7
	b1885	*tap*	methyl-accepting chemotaxis protein (TarH, HAMP & MCP domains)	SM39_2449	SMDB11_2231	99.4
	b1886	*tar*	methyl-accepting chemotaxis protein (TarH, HAMP & MCP domains)	SM39_2450	SMDB11_2232	92.6
	na	na	methyl-accepting chemotaxis protein (HAMP & MCP domains)	SM39_4406	SMDB11_4188	96.9
	na	na	methyl-accepting chemotaxis protein (HAMP & MCP domains)	SM39_4687	SMDB11_4458	94.4
	na	na	methyl-accepting chemotaxis protein (HAMP & MCP domains)	SM39_4745	SMDB11_4516	98.1

*1: domain of internal sensors of oxygen, redox potential, and light

*2: homologues of the ligand binding domain of Tar (asparate/maltose chemoreceptor)

*3: domain of histidine kinases, adenylyl cyclases, methyl binding proteins, phosphatases

na: *E. coli* K-12 contains three additional methyl-accepting chemotaxis ptoteins, trg (b1421), aer (b3072) and tsr (b4355), but their orthologues were not assigned in *S. marcescens*.

Table S7. SM39 and Db11 genes orthologous to the putative type IV pili-related genes identified/predicted in *E. coli* K-12

genes (*E. coli* K-12)	predicted functions/products (*E. coli* K-12)	SM39	Db11	amino-acid sequence identity between SM39 and Db11
hofM	pilus assembly protein	SM39_4060	SMDB11_3856	96.4%
hofN	fimbrial assembly protein	SM39_4058A	SMDB11_3855	88.8%
hofO	membrane protein	SM39_4058	SMDB11_3854	84.4%
hofP	hypothetical protein	SM39_4057A	SMDB11_3853	92.5%
hofQ	fimbrial transporter	SM39_4057	SMDB11_3852	96.9%
pppA	prepilin leader peptidase	SM39_2364*	SMDB11_2160*	91.9%
yggR	pilus retraction ATPase	SM39_3622	SMDB11_3436	96.7%
ppdA	prepilin peptidase dependent protein	SM39_3423	SMDB11_3213	91.4%
ppdB	prepilin peptidase dependent protein	SM39_3422	SMDB11_3212	93.8%
ppdC	prepilin peptidase dependent protein	SM39_3420	SMDB11_3210	96.6%
ppdD	major pilin subunit	SM39_0092	SMDB11_0093	93.1%
hofB	hypothetical protein	SM39_0091	SMDB11_0092	93.5%
hofC	assembly protein in type IV pilin biogenesis	SM39_0090	SMDB11_0091	95.2%

* Except for these genes, all *S. marcescens* genes are located at the chromosome loci analogous to those in *E. coli* K-12.

Table S8. Iron uptake systems of *S. marcescens* strains SM39 and Db11

	SM39	Db11	genes	products	Prevalence in other *Serratia* strains			
					S. plymuthica AS9	*S. plymuthica* 3Rx13	*S. odorifera* DSM4581	*S. proteamaculans* 568
**The *ent* operon and *entA*- and *entD*-homologs**								
	SM39_4670~SM39_4674	SMDB11_4411~SMDB11_4415	*entFSCEB*	enterobactin synthase subunit F, enterobactin exporter, isochorismate synthase, enterobactin synthase subunit E & isochorismatase	+	+	+	+
	SM39_2650	SMDB11_2450	-	isochorismatase (*entA* homolog)	-	-	-	-
	SM39_2652	SMDB11_2452	*pswP*	putative 4'-phosphopantetheinyl transferase (*entD* homolog)	+	+	-	+
**A gene cluster for siderophore synthesis**								
	SM39_1904~SM39_1907	SMDB11_1728~SMDB11_1731	-	predicted transporter, siderophore synthetase CbsF homolog,putative siderophore biosynthesis protein & ferric siderophore esterase	-	-	-	-
**The *fhu* operon**								
	SM39_3571~SM39_3573	SMDB11_3389~SMDB11_3391	*fhuBDC*	ferrichrome ABC transporter (permease,substrate-binding & ATP-binding proteins)	+	+	+	+
	SM39_3574	SMDB11_3392	*fhuA*	ferrichrome outer membrane transporter	+	+	-	-
**The *fep* operon**								
	SM39_3038~SM39_3041	SMDB11_2823~SMDB11_2826	*fepBDGC*	ferric siderophore ABC transporter (substrate-binding, permease & ATP-binding proteins)	-	-	-	-
**The *fec* operon**								
	SM39_1751~SM39_1753	SMDB11_1561~SMDB11_1563	*fecARI*	ferric citrate outer membrane transporter, transmembrane signal transducer & RNA polymerase sigma factor	-	-	-	-
**The *has* operon**								
	SM39_0339~SM39_0345	SMDB11_0282~SMDB11_0288	*hasIERADEB*	RNA polymerase sigma factor, putative iron sensor protein, TonB-dependent heme receptor, hemophore HasA, heme acquisition ABC transporter (ATP-binding/permease & substrate-binding proteins) & TonB-like protein	-	-	-	-
**The *hem* operon**								
	SM39_1627~SM39_1631	SMDB11_1439~SMDB11_1443	*hemVUTSR*	hemin ABC transporter (ATP-binding, permease & substrate-binding proteins), hemin-degrading protein & TonB-dependent hemin receptor	+	+	+	+
	SM39_1632	SMDB11_1444	*hemP*	hemin uptake protein	-	-	-	+
**The *hms* operon**								
	SM39_3003~SM39_3006	-	*hmsSRFH*	hemin storage system (HmsS, HmsR, HmsF & HmsH proteins)	-	-	-	-
**Other TonB-dependent receptors/transporters**								
	SM39_0195	SMDB11_0195	*iutA*	TonB-dependent iron siderophore receptor	+	+	+	+
	SM39_0333	SMDB11_0276	-	TonB-dependent receptor	-	-	+	+
	SM39_0715	SMDB11_0544	-	TonB-dependent receptor	-	-	-	+
	SM39_1900	SMDB11_1721	-	TonB-dependent copper receptor	-	-	-	+
	SM39_1908	SMDB11_1732	-	TonB-dependent ferric siderophore receptor	-	-	-	-
	-	SMDB11_1769	-	TonB-dependent receptor	-	-	-	-
	SM39_2513	SMDB11_2302		TonB-dependent heme receptor	-	-	-	-
	SM39_2582	SMDB11_2373	-	TonB-dependent receptor	-	-	-	+
	SM39_2784	SMDB11_2548	-	TonB-dependent receptor	+	+	+	+
	SM39_3043	-	-	TonB-dependent ferric siderophore receptor	-	-	-	-
	SM39_3078	SMDB11_2862	-	TonB-dependent receptor	-	-	-	-
	SM39_3117	-	-	TonB-dependent siderophore receptor	-	-	-	-
	SM39_3501	SMDB11_3301		TonB-dependent receptor	+	+	+	+
	SM39_3664	SMDB11_3469	-	TonB-dependent receptor	-	-	-	-
	SM39_4664	SMDB11_4405	-	TonB-dependent receptor	+	+	-	+
	SM39_4667	SMDB11_4408	*fepA*	iron-enterobactin outer membrane transporter	+	+	+	+
	SM39_4933	SMDB11_4677	-	TonB-dependent receptor	+	+	+	+

Table S9. The list of virulence-attenuated mutants derived from strains Db10 and Db11

Mutants	References[a]	Tn insertion sites		Genes or homologues	Known or predicted function	Conservation in SM39 (orthologs in SM39)		Conservation in other Serratia strains[b]				Notes
		Db11 CDS	Db11 coordinates			SM39	identity (aa)	*S. plymuthica* AS9	*S. plymuthica* 4Rx13	*S. odorifera* DSM4582 (draft)	*S. proteamaculans* 568	
JESM272	this study	SMDB11_0020	24359	*lysR*-like	gene regulation	SM39_0022	98.1%	+	+	-	+	
10E5	Kurz et al.	SMDB11_0276	297315		TonB-dependent ferrisiderophore receptor	SM39_0333	98.0%	-	-	+	+	
7A8	Kurz et al.	SMDB11_0595	630735	inhA	putative isonitrile hydratase	SM39_0772	96.4%	+	+	-	-	
JESM274	this study	SMDB11_0850	885287	*wza*	exopolysaccharide export	SM39_1017	86.7%	+	+	+	+	
JESM237	this study	SMDB11_0872	913110	*manC2*	O-Ag biosynthesis	-	na	(-)	(-)	(-)	(-)	SMDB11_0872 is specific to Db11 although SM39_1036 shows 73.3% sequence identity to it.<br>(-); other Serratia also contain genes showing 74-78% identity to SMDB11_0872, but they are not the ortholog of SMDB11_0872.
JESM204<br>& 20C2	this study<br>& Kurz et al.	SMDB11_0873	914362<br>& 914398	*wzm*	O-Ag biosynthesis (O-Ag ABC transporter for LPS biosynthesis)	-	na	-	-	-	-	
JESM202	this study	SMDB11_0876	918777	*wbdA*	O-Ag biosynthesis	-	na	-	-	-	-	
8C7	Kurz et al.	SMDB11_1112	1178698		T6SS effector protein of unknown function	SM39_1325	98.9%	-	-	-	-	
3H5	Kurz et al.	SMDB11_2114	2224083	*omp*	outer membrane pore protein (porin)	SM39_2321	98.1%	+	+	+	+	
18F3	Kurz et al.	SMDB11_2153	2270917	*wcaG*-like	exopolysaccharide biosynthesis	SM39_2357	98.8%	+	+	+	+	
22D9	Kurz et al.	SMDB11_2265	2387534		T6SS immunity protein	SM39_2484	91.3%	-	-	-	-	
7D1	Kurz et al.	SMDB11_2266	2387867		T6SS immunity protein	SM39_2485	89.8%	-	-	-	-	
10F7<br>& 18D4	Kurz et al.	SMDB11_2319	2468154<br>& 2468156		unknown	SM39_2525	97.5%	(+)	(+)	(+)	+	(+); 3 strains contain orthologs, but each shows 69%, 69%. 64% identity to SMDB11_2319, respectively (71% for the ortholog of S. proteamaculans 568)
8H1	Kurz et al.	SMDB11_2321	2472147	*mgtB*	Mg(2+) transport ATPase	SM39_2527	99.0%	+	+	+	+	
10H1	Kurz et al.	SMDB11_2451	2594160	*entC*	enterobactin synthetase component C	SM39_2651	98.5%	-	-	-	-	
JESM273	this study	SMDB11_2452	2594732	*entD*	enterobactin and serrawettin W2 biosynthesis	SM39_2652	93.8%	(-)	(-)	-	(-)	(-); 3 strains contain genes showing 72-74% identity to SMDB11_2452 but they are not the ortholog of SMDB11_2452.
10H4	Kurz et al.	SMDB11_2632	2783570		CPS-53 (KpLE1) prophage gene	-	na	+	-	-	-	The AS9 homologue shows 94% aa seq identity to SMDB11_2632
JESM266<br>& JESM271	this study	SMDB11_2694	2854582<br>& 2853500	*nuoG*	respiratory chain	SM39_2897	99.8%	+	+	+	+	
JESM268	this study	SMDB11_2696	2857183	*nuoE*	respiratory chain	SM39_2899	98.9%	+	+	+	+	
8E2	Kurz et al.	300 bp upstream of SMDB11_2955	3121546		SMDB11_2955: putative diacylglycerol kinase	SM39_3172	97.0%	+	+	+	+	
7F1	Kurz et al.	SMDB11_3029	3199043		unknown	SM39_3239	98.6%	-	+	-	+	
JESM270	this study	SMDB11_3042	3216592	*purL*	purine biosynthesis	SM39_3254	99.4%	+	+	+	+	
7E7	Kurz et al.	SMDB11_3228	3426079	*galR*-like	galactose operon repressor	SM39_3437	97.6%	+	+	+	+	
23C11	Kurz et al.	SMDB11_3455	3668381		T6SS-related, a minor Hcp homologue	-	na	-	-	(-)	(-)	SM39_2481 shows 66.9% identity to SMDB11_3455, but it encodes the equivalent of the main Db11 Hcp homologue, SMA2263. This is also the case for strains DSM4582 and 568 as indicated by (-).
JESM135	this study	SMDB11_3469	3684635	*fecA*-like	iron uptake	SM39_3664	98.4%	-	-	-	-	
JESM267	this study	SMDB11_3680	3915889	*swrA*	serrawettin W2 biosynthesis	-	na	-	-	-	-	SM39_3884 is present at a locus analogous to that of SMDB11_3680, but shows 55.6% amino-acid sequence identity to SMDB11_3680.
23E6	Kurz et al.	SMDB11_3684	3925480	*yjcE*-like	unknown , putative cation/H+ antiporter	SM39_3888	99.8%	+	+	+	+	
21C4	Kurz et al.	SMDB11_3736	3978018	*shlBA*	hemolysin production	SM39_3935	97.9%	+	+	-	+	
22D4	Kurz et al.	SMDB11_4162	4447091	*ibpB*	chaperone IbpB	SM39_4435	100.0%	+	+	+	+	
8G1	Kurz et al.	SMDB11_4219	4511266		two-component system sensor protein	-	na	-	-	-	-	
21C1	Kurz et al.	SMDB11_4556	4902585		ATPase similar to R64 plasmid IbfA involved in growth inhibition of phage B23	-	na	-	-	-	-	

[a] Mutants indicated by "Kurz et al." were identified in a previous screening using Db11 and solid media (Kurz et al., EMBO J, 22:1451-1460, 2003).

[b] The threshold used in this analysis was >70% amino-acid sequence identity and >70% overlap. See the comments for the genes indicated by (-) and (+).
